# The Chemical Composition, Pharmacological Activity, Quality Control, Toxicity, and Pharmacokinetics of the Genus *Clinopodium* L.

**DOI:** 10.3390/molecules30112425

**Published:** 2025-05-31

**Authors:** Wen Li, Jianping Pan, Xiaobing Chen, Senhui Guo, Xilin Ouyang

**Affiliations:** Department of Pharmacy, Gannan Health Vocational College, Ganzhou 341000, China; 18270736936@163.com (W.L.); pan_jianping@gnhvc.edu.cn (J.P.); baiihrgw11@163.com (X.C.); gshyh2003@126.com (S.G.)

**Keywords:** *Clinopodium* L., phytochemistry, pharmacology, quality control, toxicity, pharmacokinetics

## Abstract

The genus *Clinopodium* L. (Lamiaceae) comprises perennial herbaceous plants known for their diverse pharmacological properties. Clinically, these plants are mainly used for the treatment of various hemorrhagic disorders. This review systematically summarizes the research progress on the chemical composition, pharmacological activity, quality control, toxicity, and pharmacokinetics of the genus *Clinopodium* by searching Google Scholar, Scopus-Elsevier, Wiley, Springer, Taylor & Francis, Medline, Web of Science, CNKI, Weipu, Wanfang, and other academic databases over the last decade (March 2015–February 2025). To date, more than one hundred and thirty structurally diverse secondary metabolites have been isolated and identified from this genus, including flavonoids, triterpenoid saponins, diterpenoid glycosides, lignans, and phenylpropanoids. In addition, numerous volatile oil constituents have been identified in over forty species of the genus *Clinopodium*. Crude extracts and purified compounds exhibit a variety of pharmacological activities, including hemostatic, anti-myocardial cell injury, cardiovascular protective, anti-inflammatory, antimicrobial, antitumor, hypoglycemic, and insecticidal properties. However, current quality assessment protocols in the genus *Clinopodium* are limited to flavonoid- and saponin-based evaluations in *C. chinense* (Benth.) O. Kuntze and *C. gracile* (Benth.) O. Matsum. Further research is needed to elucidate the pharmacological mechanisms, toxicity, and possible interactions with other drugs. Therefore, the genus *Clinopodium* has a wide range of biologically active compounds with potential applications in drug development for hemostasis and cardiovascular protection. Nevertheless, there is also an urgent need to establish standardized methodologies to address uncertainties concerning the safety and efficacy of injectable extracts or compounds.

## 1. Introduction

The genus *Clinopodium* L. is a class of perennial herbaceous plants in the Lamiaceae family. They are widely distributed in Asia and Europe, typically growing in wet areas, such as fertile forest margins, stream banks, and river valley grasslands. The Lamiaceae family is highly diverse, and the generic boundaries of the genera *Satureja*, *Micromeria*, *Clinopodium*, and *Acinos* are unclear [1]. Of these, *Satureja* and *Micromeria* are accepted genera, while *Calamintha* and *Acinos* are considered synonyms of *Clinopodium* following a comprehensive taxonomic revision and phylogenetic study of Old World *Clinopodium* [2]. Currently, there are approximately 192 *Clinopodium* species worldwide [3].

**Figure 1:**
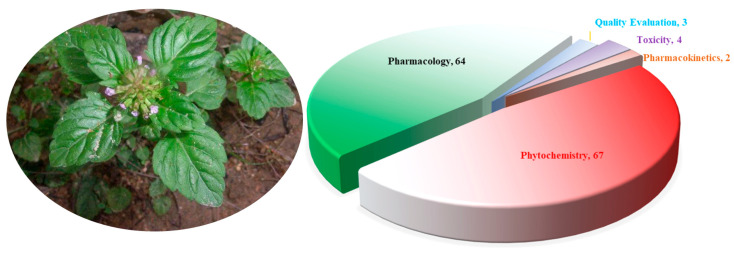
Article distribution of the genus *Clinopodium* L.

The morphological differences between species in the genus *Clinopodium* are relatively minor. Plants in this genus often have creeping stems that are finely longitudinally striate and densely glandular pubescent. They have ovate, opposite leaves with crenate serrations. They also have verticillasters, with narrowly tubular calyxes and purple-red corollas. The apex of the upper lip of the corolla is emarginate. Most *Clinopodium* plants flower from April to May and bear fruit from May to June. The nutlets are dark brown, oblong/oval, and smooth or pimply. They have a faint scent and an astringent, slightly bitter taste. The genus *Clinopodium* contains various pharmacologically active compounds, including terpenoids, phenylpropanoids, flavonoids, polyphenols, and fatty acids. This genus is also rich in essential oils [4]. Plants of *Clinopodium* have long been used in folk medicine to prevent and treat various diseases due to, for example, their hemostatic, blood circulation, uterine contraction, antibacterial, anti-inflammatory, immunomodulatory, hypoglycemic, endothelial cell protection, antitumor, sedative, hypnotic, analgesic, and anticonvulsant properties [5,6]. They have been proven to positively affect the cardiovascular system, and to clinically cure gynecological bleeding and oral bleeding diseases. They also have positive effects on hereditary telangiectasia, allergic purpura, and simple purpura. To better discover the current state of research on the genus *Clinopodium*, electronic databases (Google Scholar, Scopus-Elsevier, Wiley, Springer, Taylor & Francis, Medline, Web of Science, CNKI, Weipu, Wanfang, and other academic databases) were extensively searched using keywords including “*Clinopodium*”, “*Calamintha*”, “*Acinos*”, and “Fengluncai” (the Chinese name: 风轮菜). Over the past decade, a total of 119 articles on the chemical composition, pharmacological activity, quality control, toxicity, and pharmacokinetics of the genus *Clinopodium* were retrieved (Figure 1).

This review summarizes the results of research conducted over the past decade on the phytochemistry and activity of *Clinopodium* plants. This research can provide a basis for developing drugs derived from *Clinopodium* plants.

## 2. Phytochemistry

From March 2015 to February 2025, a total of one hundred and thirty-six isolated and purified compounds were reported from the genus *Clinopodium*. These compounds included sixty-seven triterpenoid saponins, twenty-six flavonoids, fifteen phenylpropanoids, and sixteen uncategorizable compounds (compound names are shown in Table 1). These structures were elucidated using infrared spectroscopy (IR), mass spectroscopy (MS), and nuclear magnetic resonance (NMR, including ^1^H NMR, ^13^C NMR, COSY, NOESY, HSQC, 2D TOCSY, HSQC-TOCSY, and HMBC) analysis, as well as electrochemical circular dichroism (ECD) and data reported in the literature. For the new compounds containing the sugar moiety, the type and absolute configuration of the sugars were also determined by acid hydrolysis. In addition, most of the monoterpenes and sesquiterpenes were identified from essential oils (EOs), including piperidone oxides and piperidone epoxides. The LC-MS methodology was also employed to investigate the compounds. It is worth noting that some previously isolated compounds, including naphthoquinones and fatty acids, were not identified as new constituents during this period [7].

### 2.1. Compounds Isolated and Identified from the Genus Clinopodium

#### 2.1.1. Terpenoids

**Figure 2:**
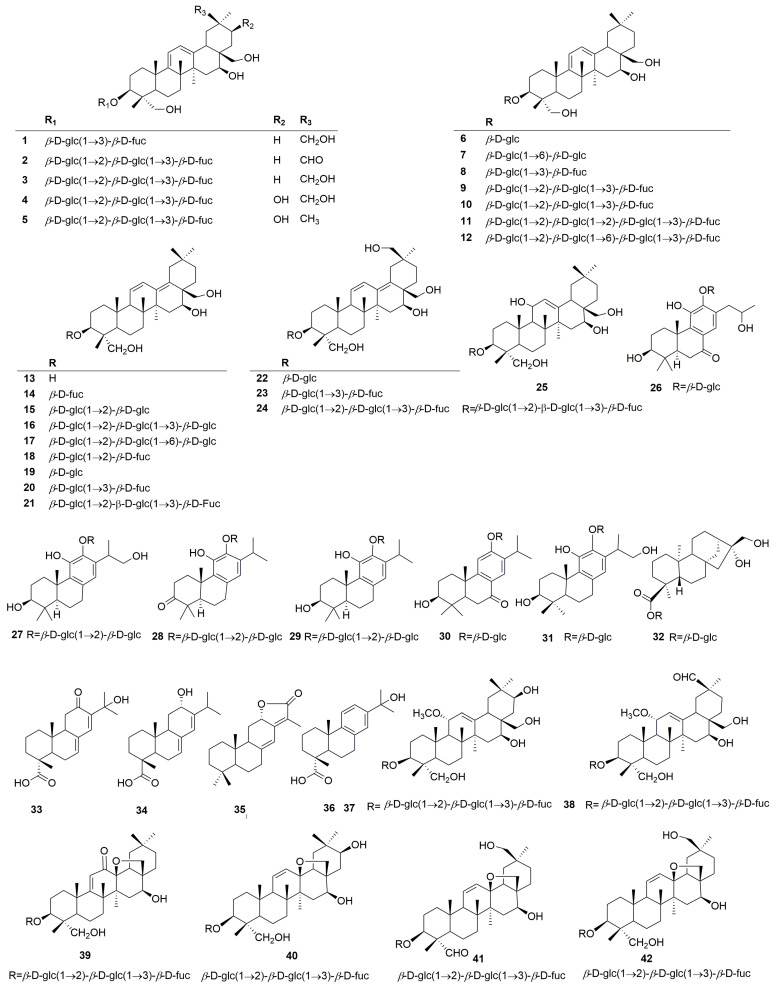

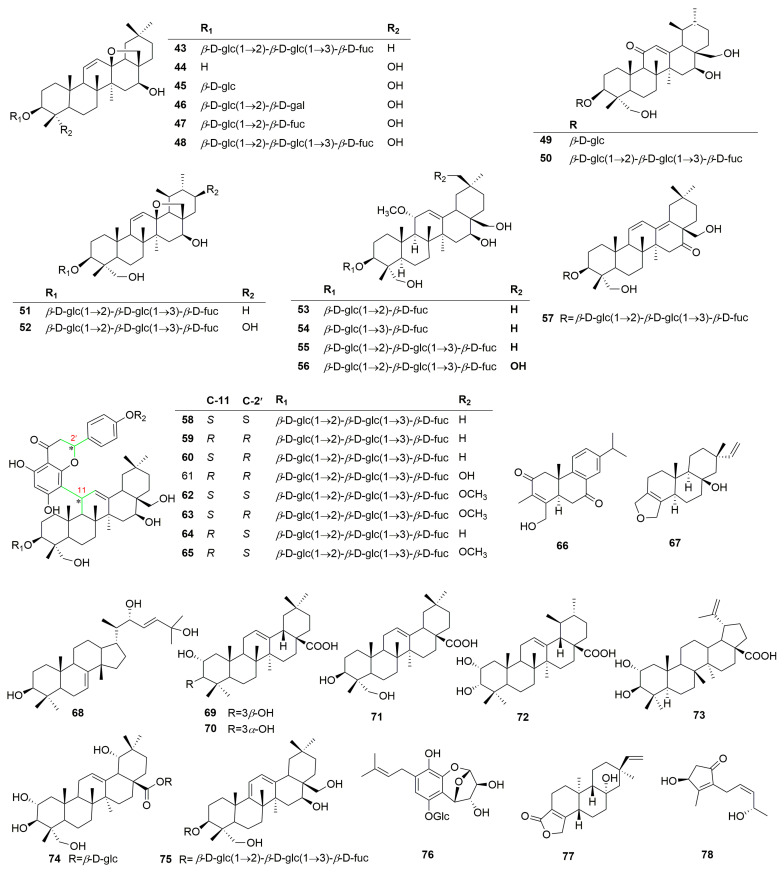
Structures of terpenoids or their saponins from the genus *Clinopodium*.

Terpenoids are the main active constituents of the genus *Clinopodium*. Seventy-eight terpenoids or their saponins have been isolated from plants of this genus, including thirty-six new compounds. Figure 2 shows the structures of the terpenoids and their saponins. The main structures of triterpenoids are oleanane, ursane, lupine, and saikosaponin (containing 13, 28-oxygen rings) skeletons. In addition to the triterpenes, there are fourteen diterpenoids and one monoterpenoid. Most terpenoids exist as glycosides. The sugar composition is mainly glucose, fucose, and rhamnose, and the number of sugars ranges from one to five, typically two to three. Of the seventy-eight terpenoids that were isolated and identified, fifty-one were found in *C. chinense*, seventeen in *C. polycephalum*, fourteen in *C. gracile*, four in *C. bolivianum*, and two in *C. umbrosum*. This suggests that *C. chinense*, *C. polycephalum*, and *C. gracile* are used more frequently in developing applications. Furthermore, the simultaneous isolation of buddlejasaponin IV (**48**), buddlejasaponin IVa (**55**), and buddlejasaponin IVb (**21**) from *C. gracile*, *C. chinense*, *C. umbrosum*, and *C. polycephalum* indicates that these compounds may be the characteristic components of the genus *Clinopodium*. These saponins were mostly extracted from the whole herb or the plant’s aerial parts using 70–80% ethanol as the extraction solvent.

#### 2.1.2. Flavonoids

**Figure 3:**
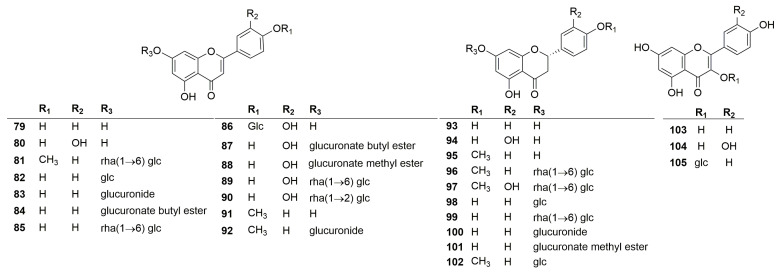
Structures of flavonoids from the genus *Clinopodium*.

A total of twenty-seven flavonoids have been identified in the plants of the genus *Clinopodium*. Their chemical structures are shown in Figure 3. According to the structure of the aglycone, the flavonoids are mainly divided into three categories, including flavonoids (**79**–**92**), dihydroflavonoids (**93**–**102**), and flavonols (**103**–**105**). Among these compounds, polycephalum B (**101**) is a new compound derived from *C. polycephalum* [19], and the rest were obtained from *C. chinense*. In addition, most of these compounds are derivatives of luteolin, quercetin, and kaempferol. Notably, an artificial compound, luteolin 7-*O*-*β*-D-glucuronide butyl ester (**82**), was incorrectly written as apigenin 7-*O*-*β*-D-pyranglycuronate butyl ester [26].

#### 2.1.3. Phenylpropanoids

**Figure 4:**
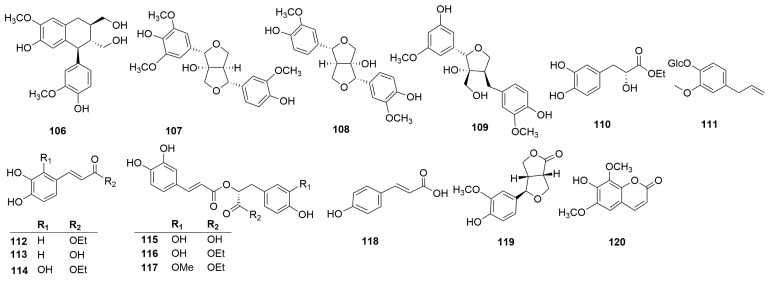
Structures of phenylpropanoids from the genus *Clinopodium*.

Phenylpropanoids are natural compounds consisting of a benzene ring linked to three straight-chain carbons (C6-C3 groups). Phenylpropanoids mainly include phenylpropionic acid, lignin, and coumarin. Thirty phenylpropanoids have been isolated and identified from *C. chinense* (Figure 4). Xu et al. isolated four lignin compounds—(+)-isolariciresinol (**106**), fraxiresinol (**107**), 8-hydroxy-7′-epipinoresinol (**108**), and deltoignan A (**109**)—as well as a phenylpropanoid compound salicifoliol (**119**) and a coumarin isofraxidin (**120**) from *C. chinense* [12]. Zeng et al. obtained the following compounds from *C. chinense*: ethyl (2*R*)-3-(3,4-dihydroxyphenyl)-2-hydroxypropanoate (**110**), ethyl (2*E*)-3-(3,4-dihydroxyphenyl)-prop-2-enoate (**112**), caffeic acid (**113**), ethyl (2*E*)-3-(2,3,4-trihydroxyphenyl)-prop-2-enoate (**114**), ethyl rosmarinate (**116**), and clinopodic acid B (**117**) [25]. Wang et al. identified two phenylpropionic acids named caffeic acid (**113**) and *p*-hydroxycinnamic acid (**112**) [26].

#### 2.1.4. Other Compounds

**Figure 5:**
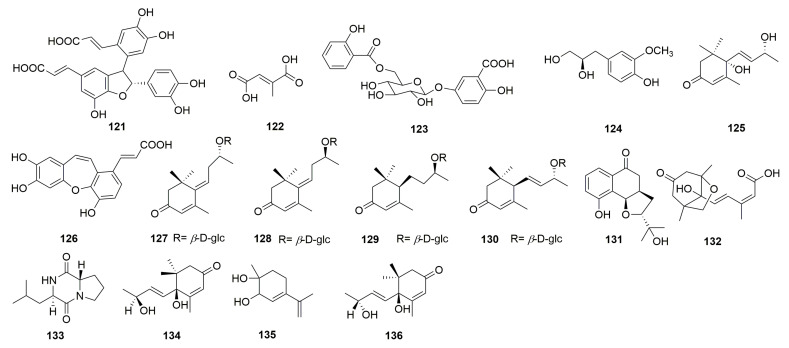
Structures of other compounds from *the genus Clinopodium*.

In addition, sixteen other compounds were discovered from *C. chinense*, including *cis*-3-[2-[1-(3,4-dihydroxy-phenyl)-1-hydroxymethyl]-1,3-ben-zodioxol-5-yl]-(*E*)-2-propenoic acid (**121**), mesaconic acid (**122**), gentisic acid 5-*O*-*β*-D-(6′-salicylyl)-glucopyranoside (**123**) [26], 4-hydroxyl-3-methoxyphenyl-1-propane-1,2-diol (**124**) [12], blumenol A (**125**) [12], tournefolic acid B (**126**) [27], (*E*)-6-[9*R*-(*β*-D-glucopyranosyloxy) butylidene]-1,1,5-trimethyl-4-cyclohexen-3-one (**127**), (*E*)-6-[9*S*-(*β*-D-glucopyranosyloxy) butylidene]-1,1,5-trimethyl-4-cyclohexen-3-one (**128**), blumenol C 9-*O*-*β*-D-glucopyranoside (**129**), (6*R*,9*R*)-3-oxo-*α*-ionol-9-*O*-*β*-D-glucopyranoside (**130**) [23], and Chinense B (**131**) (Figure 5). Compounds **121** and **122** are a pair of isomers and compounds **127** and **128** are known megastigmane-type glycosides. Chinense B (**131**) is a rare compound with a ringed prenylated naphthoquinoid skeleton [23]. Phaseic acid (**132**), *cyclo*-(*S*-Pro-*R*-Leu) (**133**), vomifoliol (**134**), *p*-mentha-3,8-dien-1,2-diol (**135**), and corchoionol C (**136**) were isolated and purified from the *n*-butanol extract of *C. chinense* [24].

### 2.2. Compounds Identified by GC-MS

The genus *Clinopodium* represents a rich source of chemically diverse essential oils (EOs) with significant pharmacological potential [28]. Gas chromatography–mass spectrometry (GC-MS) combined with a computerized search is an important tool for the analysis and identification of EOs. More than forty articles have reported on the chemical composition of EOs from nearly thirty *Clinopodium* species (Table 2), indicating that genus *Clinopodium* EOs are a popular research topic.

As can be seen from Table 2, the chemical composition of genus *Clinopodium* EOs is predominantly characterized by monoterpenes, oxygenated monoterpenes, sesquiterpene hydrocarbons, and oxygenated sesquiterpenes, such as pulegone, menthone, piperidone oxide, menthol, piperidone, *E*-caryophyllene, *α*-pinene, and their derivatives, which serve as dominant markers [29,30,31]. EOs extracted from different species in various regions of the globe reveal that the chemical type of EOs depends on the species category, geography, extraction solvent, collection time, and other factors [32,33,34]. EOs extracted from *S. calamintha nepeta* with water vapor contain high concentrations of 1,8-cineole (34.34%) and *cis*-pinene (11.87%) [35], while those extracted with supercritical CO_2_ contain high concentrations of piperone oxide [36]. Although the compositions of the same species were somewhat similar, their content varied greatly [37]. Environmental stressors, such as temperature and UV exposure, direct terpene biosynthesis. Cultivation introduces additional variability. For instance, wild *S. calamintha* has higher levels of pulegone (72.93% vs. 68.58% in cultivated plants [38]), which suggests that domestication may dilute certain bioactive compounds. Geographical origin profoundly shapes EO composition, often overriding phylogenetic boundaries. Mediterranean species (e.g., *C. nepeta* from Italy [39]) accumulate 1,8-cineole (34.09%) and eugenol (14.66%), whereas alpine populations (e.g., *C. nubigenum* from Ecuador [40]) prioritize carvacrol (32.9%) and pulegone (25.4%). *C. rouyanum* from the Mountains of the island of Majorca in Spain, belongs to both Mediterranean species and alpine populations, are pulegone-dominant, containing 73–82.2% pulegone [41]. Altitudinal shifts also drive variability. *C. thymifolium* from Serbia exhibits a decrease in pulegone content from 75.9% at low elevations to 50.4% in the mountains, alongside an increase in isomenthone content from 3.1% to 17.8% [42].

It is worth noting that terpinene-4-ol, *α*-terpineol, carvone, isopiperitenone, 4-hydroxyisopiperitenone, and 4-hydroxypiperitone were obtained from limonene, as well as that 8-hydroxythymol was obtained from thymol by biotransformation. Piperitenone, diosphenolene, and 1-hydroxy-*p*-menthan-3-one emerged as artifacts due to the opening of the epoxide rings in acidic conditions caused by fungi from piperitenone- and piperitone-epoxides, respectively [43].

**Table 2:** Chemical constituents of genus *Clinopodium* EOs.

No.	Name	Collected Areas	Identified Compounds and Major Components	Ref.
1	*C. axillare*	Quiriría, San José, Province of Esteban Arce	Seventy-five compounds were identified. The major constituents were piperidone oxide (20–30%), piperidone epoxide (15–19%), piperidone (13%), pulerone (3–5%), and piperidone (4–5%), as well as limonene (8–12%) and -pinene (1–5%).	[37]
2	*C. brevicalyx*	The southern high Andean regions of Peru	The most abundant compounds were isomaltone (44.25%), menthol (22.22%), and prunone (8.23%).	[44]
3	*C. brownei*	Amazonian region of Ecuador	Non-polar constituents: ethyl cinnamate (21.4%), pridon (20.76%), methyl cinnamate (16.68%), caryophyllene (8.17%), *β*-chromoselenene (7.92%), and menthone (7.51%).Polar constituents: pridon (29.90%), ethyl cinnamate (18.75%), methyl cinnamate (13.82%), caryophyllene (10.0%), and menthone (8.04%).	[45]
4	*Calamintha baborensis*	Jijel eastern region of Algeria	The major constituent is eugenol (27.04%), followed by 3-methoxy acetophenone (26.4%) and phenyl ethyl alcohol (6.58%).	[46]
5	*C. candidissimum*	Region of Djebel Murdjadjo, Oran, Northwestern Algeria	Thirty-eight compounds were identified, including oxygenated monoterpenes pulegone (44.8%), piperitenone ^1,^ * (6.6%), isopulegone (5.8%), and neo-menthol (3.8%). Among them, the sesquiterpene hydrocarbons germacrene D (16.2%) and bicyclogermacrene (3.0%) were the most abundant.	[47]
6	*C. chinense*	Lishui, Zhejiang Province, China	Thirty-five compounds were identified, accounting for 99.18% of the total oil. The major components were phorbol (18.54%), piperitone (18.9%), caryophyllene (12.04%), and bornyl acetate (8.14%), followed by caryophyllene oxide (4.19%), piperitone (4.09%), and carvacrol (4.01%).	[48]
7	*Satureja calamintha*	Faculty of Sciences Semlalia, Marrakech, Morocco	Fifteen compounds accounted for 99.88% and 98.14% of the total oils obtained from wild and cultivated plants, respectively. Pulegone (72.93–68.58%), menthone (12.07–10.15%), and menthol (6.31–9.83%) were found as the main constituents.	[38]
8	*Satureja calamintha*	Taounate, Morocco	Twenty-four compounds were identified. The main constituents were pulegone (21.48%), piperitenone * oxide (17.71%), and eucalyptol (11.99%).	[49]
9	*Satureja calamintha*	Jijel region of Algeria	Three most abundant compounds identified were l-menthone (32.10%), *neo*-menthol (32.07%), and pulegone (22.35%).	[50]
10	*Calamintha fenzlii*	Nablus region of Palestine	The chemical constituents were dominated by oxygenated monoterpenoid (96.91%). The major chemical components were represented by menthone 68.93% and pulegone 23.1%.	[51]
11	*Calamintha glandulosa*	Luštica in Stari Krašići (Montenegro)	Seventeen compounds were identified. The major compounds were pulegone (35.1%), piperitenone * (23.4%), menthone (15.7%) and piperitone (11.5%).	[30]
12	*Calamintha incana (Sm.) Boiss.*	Kestel, Bursa, Turkey	The oxygenated monoterpenes *trans*-piperitone oxide (41.37%), piperitenone oxide (34.47%), piperitenone * (6.67%), and monoterpene phenol thymol (3.37%) were found to be the major constituents.	[52]
13	*Calamintha incana*	Ajloun county in Jordan	The main constituents were benzenamine-4-methyl-3-nitro-(34.11%) and (2S,4R)-*p*-mentha-6,8-diene 2-hydroperoxide (31.48%).	[53]
14	*C. macrostemum*	San Andrés, Paxtlán, Oaxaca, México	Twenty-six compounds were identified, including menthone (approximately 35%) and piperitone oxide (approximately 30%).	[54]
15	*C. menthifolium*	AinDraham, Babouch, and Tabarka, Tunisia	Sixty-three different compounds were identified: piperitone (34.5%), *cis*-piperitone oxide (26.1%), and piperitone (47.9%).	[55]
16	*Calamintha nepeta*	Vratarnica near Zaječar (Serbia)	Fourteen compounds were identified. The major compounds were pulegone (58.0%) and piperitenone * (27.4%).	[30]
17	*Calamintha nepeta*	Morano Calabro, Cosenza, Italy	Thirty-four compounds were identified. The major components were 1,8-cineole (34.09%), eugenol (14.66%) and linalool acetate (11.25%), followed by sabinene (6.97%) and linalool (6.64%).	[39]
18	*Calamintha nepeta*	Beni-Saf region in the northwest of Algeria	The primary components included oxygenated monoterpenes, notably pulegone (58.36%), isoborneol (10.40%), menthone (8.91%), and piperitenone * (3.86%).	[56]
19	*Calamintha nepeta*	Basilicata region, Southeastern Italy	Twenty-four compounds were identified, accounting for 90.17% of total oil composition. Pulegone (44.7%), menthone (16.4%), piperitenone * (13.3%), and piperitone (6.01%) were the major constituents.	[57]
20	*Calamintha nepeta*	Tarquinia, Viterbo, Italy	Thirty-nine different chemical constituents have different concentrations in various fractions. Pulegone (37.7–77.7%) and crysanthenone (14.4–27.3%) were the most abundant components.	[32]
21	*Calamintha nepeta*	Alentejo region, Herdade da Mitra, Évora	Twenty-nine compounds were identified, representing 91% of oxygenated monoterpenes, 7% of hydrocarbon monoterpenes, and 1% of sesquiterpenes. The major components were 1,8-cineole (28%), menthone (22%), menthol (16%), and pulegone (5%).	[58]
22	*Calamintha nepeta*	Tengalti village and the region near the Velvelechay river of Quba	Seventy-eight compounds were identified; the major components were thymol (19.81%), cyclopropane, 1,1-diethyl-(19.77%), cyclohexanone, 3-vinyl3-methyl-(18.66%), D-limonene (7.45%), and caryophyllene (6.16%).	[59]
23	*Satureja calamintha subsp. nepeta Briq.*	Medea region, South Algiers and Chlef region, western Algiers	Seventy compounds were identified, representing 97.4% of the oil. 1,8-cineole (28.4%), pulegone (10.2%), menthone (9.7%), and isomenthone (9.6%) were the most important constituents.	[60]
24	*S. calamintha nepeta*	Mountains of the Skikda region located in northeastern Algeria	One hundred and ten compounds were identified. Piperitenone oxide, *trans*-piperitenone oxide, caryophyllene oxide, 3-methyldiphenyl ether, (*E*)-caryophyllene, gensmin, germacrene D, (*Z*)-jasmone, *trans*-calamenene, *γ*-gurjunene, and pulegone are the main constituents.	[61]
25	*S. calamintha nepeta*	Mountainous terrain of the Moroccan province of Ouazzane	Twenty-seven compounds were identified, making up 99.2% of the essential oil, with 1,8-cineole (34.34%) and *cis*-pinocamphone (11.87%) being the most significant.	[35]
26	*C. nepeta*	Béni-Mtir (Aîn Draham, Jendouba), North-western Tunisia	Forty-seven compounds were identified: the main components were piperitone oxide (16.3–51.7%) and piperitenone oxide (23.4–39.3%).	[33]
27	*C. nepeta*	Bilecik, Turkey	Forty-four compounds were identified. The main components were piperitenone oxide (47.8%), limonene (18.6%), and piperitone oxide II (13.6%).	[62]
28	*C. nepeta*	Antalya-Finike, in southwestern Turkey	Thirty-five compounds were identified and quantified. The major compounds were sabine (34.2%), *β*-pinene (25.9%), *α*-pinene (13.8%), and caryophyllene oxide (3.7%).	[63]
29	*C. nepeta*	sub-Mediterranean area of Bosnia and Herzegovina	The EOs contained 42 compounds, including pulegone (44.8%), piperitenone * (48.8%), and piperitenone oxide (60.2%) as the major compounds.	[64]
30	*C. nubigenum*	Mountains near Hacienda Zuleta, Imbaburra, Ecuador	Thirty-three compounds were identified. The major chemical constituents were carvacrol (32.9%), followed by pulegone (25.4%). Other important volatiles were *p*-cymene (9.1%) and *iso*-menthone (6.4%). Monoterpenes, both in their oxygenated and hydrocarbon forms (74 and 19.7%, respectively), were the major chemical class.	[40]
31	*Calamintha officinalis*	Northern Iran (Guilan, Lahijan)	Forty-one components were isolated, constituting 23.09% of the total oil. The major constituents were *trans*-caryophyllene (8.55%), isomenthol (2.98%), tetrahydrolinalyl acetate (2.96%), and pinene (2.24%).	[65]
32	*C. pulegium*	Svrljiški Timok gorge, Serbia	Nineteen previously described mono- and sesquiterpenes were found. The major compound was menthone (47.1%), followed by *β*-pinene (19.8%), isomenthone (12.3%), and pulegone (12, 8.5%).	[66]
33	*C. rouyanum*	Mountains of the island of Majorca, Spain	Twenty-seven compounds were identified from five samples of *C. rouyanum*, among which pulegone (73.0–82.2%), menthone (6.5–11.8%), and limonene (3.5–6.0%) were the major compounds.	[41]
34	*C. serpyllifolium*	BERC Experimental Station, Til, Nablus, Palestine	Twenty-three compounds were identified. Pulegone (50.22–81.51%), menthol (1.91–15.68%), and *p*-menth-3-en-8-ol (1.64–11.94%) were the major compounds.	[67]
35	*C. serpyllifolium*	The Newe Ya’ar living germplasm, Israel	The major constituents were oxygenated monoterpenes pulegone (10.4–50.6%), piperitenone oxide (3.2–28.6%), piperitenone * (0.9–14.6%), *trans*-piperitone oxide (0.3–11.2%), iso-menthol (0.3–8.8%), and sesquiterpene *β*-caryophyllene (7.4–13.7%).	[68]
36	*C. sericeum*	Region of Cajamarca (Perú)	Seventy-three compounds were identified. The major compounds were *β*-germacrene D (15%), *β*-caryophyllene (13.8%), and sabinene (11.2%).	[69]
37	*Calamintha sylvatica*	Morano Calabro, Cosenza, Italy	Twenty compounds were identified. The major compounds were piperitone oxide (37.70%), pulegone (20.91%), and piperitenone oxide (18.26%), *iso*-menthone (7.5%), and limonene (6.58%).	[39]
38	*Calamintha sylvatica*	The edge of a beech and hornbeam forest, under Mt. Rudnik (Serbia)	Twenty-eight compounds were identified. The major compounds were *cis*-piperitone epoxide (63.3%) and menthone (10.8%).	[30]
39	*C. taxifolium*	Province of Loja, Mount Villonaco	Thirty-seven compounds were identified, mainly including (*E*)-*β*-caryophyllene (17.8%), *α*-copperene (10.5%), *β*-bourbonene (9.9%), *δ*-carpentene (6.6%), *cis*-cadina-1(6),4-diene (6.4%), and myricene D (4.9%).	[70]
40	*C. thymifolium*	Limestone habitat near Tutin, SW Serbia	Fifty-six compounds were identified, mainly including pulegone (75.9% in vegetative stage and 50.4% in late flowering stage), piperitenone * (6.2% in vegetative stage and 10.4% in late flowering stage), isomenthone (3.1% in vegetative stage and 17.8% in late flowering stage), and limonene (vegetative stage).	[42]
41	*C. umbrosum*	Kheyroud forest near Noshahr, Mazandaran, Iran	Sixteen compounds were identified. The major compounds were tolualdehyde (29.16%), palmitic acid (17.57%), and acetophenone (13.44%).	[18]
42	*Calamintha vardarensis*	The underbrush in Radika Canyon (FYR Macedonia)	Twenty-five compounds were identified. The major compounds were pulegone (51.6%) and menthone (19.9%)	[30]

^1^ Compounds with * are artifacts.

### 2.3. Compounds Identified by LC-MS

High-performance liquid chromatography–Orbitrap high-resolution mass spectrometry (UHPLC-HRMS) is widely used in plant metabolite studies because of its high sensitivity, accuracy, and rapidity. The constituents of *Clinopodium* plant extracts were easily identified using the UHPLC-Q-TOF-MS methodology.

Twenty-five compounds, including twelve flavonoids, nine saponins, and four organic acids, were identified in different batches of *C. chinense*, such as didymin (**96**), hesperidin (**97**), kaempferol (**103**), quercetin (**104**), naringenin (**93**), apigenin (**79**), saikosaponin a (**46**), buddlejasaponin IVb (**21**), acacetin (**91**), and isosakuranetin (**95**) [71]. Cluster analysis of these major constituents revealed similarities among *C. chinensis*, and those analyzed by LC-MS were consistent with the isolated and identified compounds. In contrast, the ethanolic or methanolic extracts of *C. incana* predominantly contained linolenic acid, myristic acid, and *p*-cymene [72,73]. These fatty acids accounted for 31.3% of the identified compounds (31.3%), followed by sesquiterpenes (23.9%) and monoterpenes (20.7%).

The major compounds identified in *C. nepeta* were caffeic acid (**113**), quercetin (**104**), and rosmarinic acid [29]. Similar organic acid constituents were also found in *C. vulgare*. LC-MS analysis revealed hundreds of compounds in *C. vulgare*, including organic acids such as coumaric acid and chlorogenic acid, as well as a high content of triterpene saponins [74]. Additionally, *C. vulgare* extracts also contained flavonoids and monomers, dimers, trimers, and tetramers of caffeic acid, as detected in both lyophilized aqueous and methanol extracts [75,76]. The relative abundance of caffeic acid derivatives was closely related to the harvest time. Plants collected in winter contained nearly four times more acetoacetic acid and its derivatives than they did caffeic acid derivatives [77].

Therefore, the genus *Clinopodium* contains three major classes of bioactive constituents: polyphenols, flavonoids, and saponins. These compounds are key markers for both pharmacological activity screening and quality control assessments. They provide a reliable chemical basis for evaluating the therapeutic potential and standardization of *Clinopodium* species.

## 3. Pharmacology

The various species of the genus *Clinopodium* have very rich chemical compositions, resulting in numerous pharmacological activities. These activities mainly include hemostatic, anti-myocardial cell injury, cardiovascular protection, anti-inflammatory, antimicrobial, antitumor, hypoglycemic, and insecticidal properties (Table 3).

### 3.1. Hemostatic Activity

*C. chinense* and *C. polycephalum* exhibited significant hemostatic activity and are considered legitimate sources of “Duan Xue Liu” [105]. *C. chinense* extract primarily promoted platelet adhesion, shortened plasma recalcification and bleeding times, and reduced bleeding volume [78]. Investigating the mechanism by which *C. chinense* extract reduced uterine bleeding revealed that it inhibits the inflammatory response and promotes endometrial repair. The extract also increased microvessel density (MVD) and the levels of thromboxane B2 (TXB2), vascular endothelial growth factor (VEGF), and transforming growth factor-beta (TGF-β). Additionally, it also reduced the levels of interleukin-6 (IL-6)/tumor necrosis factor-alpha (TNF-α) and the expression of matrix metalloproteinases (MMPs) 2/9. These effects promote endometrial recovery [79]. Total saponins of *C. chinense* increased coagulation function and promoted endometrial repair when used to treat functional uterine bleeding. They also significantly increased TXB2 levels and the TXB2/6-keto-PGF_(1*α*) ratio. They also increased progesterone and FSH levels.

Similarly, total flavonoids were found to significantly increase E_2_, FSH, and LH levels. They also regulated estrogen levels and reduced inflammatory responses. Total flavonoids significantly decreased the amount of uterine bleeding, reduced pathological damage to the endometrium, and increased microvessel density in the endometrial tissue. Total extracts have the best therapeutic efficacy, revealing the synergistic effects of Chinese medicine on abnormal uterine bleeding [80]. Further research confirmed that buddlejasaponin IVb (**21**), hesperidin (**97**), naringenin (**93**), apigenin (**79**), and saikosaponin a (**46**) were the major active constituents in *C. chinense* responsible for reducing uterine bleeding [71]. In addition, buddlejasaponin IV (**48**) and prosaikogenin A (**14**) from *C. chinense* can significantly promote platelet aggregation, with EC_50_ values of 53.4 and 12.2 μM, respectively. Buddlejasaponin IVb (**21**) and saikogenin F (**45**) at 200 μM shortened TT by 20.6% and 25.1%, respectively [11]. However, the methanolic fraction of *C. officinalis* has an antithrombotic effect in experimental model mice. Further detailed phytochemical and pharmacological studies are necessary to identify the antithrombotic compounds and their mechanism of action [106].

### 3.2. Anti-Cardiomyocyte Damage and Cardiovascular Protection

Flavonoids are important metabolites that can improve cardiovascular risk factors [107,108]. Total flavonoids from *C. chinense* exert myocardial protective effects through multi-target mechanisms. Studies have shown that total flavonoids from *C. chinense* can significantly increase the activity of antioxidant enzymes such as SOD, CAT, and GSH-Px, while decreasing the levels of oxidative damage markers MDA and LDH. They can also enhance cardiomyocytes by activating the nuclear translocation of Nrf2 and the downstream expression of HO-1 antioxidant defense [81,82]. Total flavonoids from *C. chinense* inhibited the expression of pro-apoptotic factors p53, caspase-3, and P21. They also blocked the phosphorylation of oxidative stress-related MAPK signaling pathways (JNK, p38, and ERK), and activated the PI3K/AKT pathway, thereby increasing the viability of H9c2 cardiomyocyte [83]. Further studies have demonstrated that *C. chinense* total flavonoids also attenuate hypoxia/reoxygenation-induced cardiomyocyte injury by inhibiting miR-702-5p expression in concert with miRNA inhibitors [84].

Monomeric compounds were obtained for further screening of cardioprotective activity. The cell viabilities of clinopodiside X (**3**), clinopodiside XI (**4**), clinoposaponin XIX (**42**) (at 50.0 μg·mL^–1^), clinopoditerpene B (**26**) (at 12.5 μg·mL^−1^), and prunin (**98**) (at 25.0 mg·L^–1^) isolated from *C. chinense* were 78.46%, 80.77%, 79.55%, 73.7%, and 84.25%, respectively. These results suggest that these compounds have cardioprotective effects against A/R or H_2_O_2_-induced apoptosis in H9c2 cells [8,14,26]. Similarly, the terpene constituents of *C. polycephalum*, clinopodiside VI (**6**), saikosaponin c (**18**), and arjunglucoside I (**74**) increased cell viability by 77.8%, 80.9%, and 79.8% at 100.0 μg·mL^−1^, respectively, showing a moderate inhibitory effect on H_2_O_2_-induced H9c2 cell damage [9]. Clinoposides G (**53**) and H (**54**), two new flavonoid–triterpenoid saponin subterpenoids from *C. chinense*, significantly increased mitochondrial membrane potential, increased antioxidant enzyme activities, decreased inflammatory cytokine levels, decreased p65 protein levels, and increased nuclear Nrf2 levels, which inhibited hypoxia/reoxygenation (A/R)-induced injury and apoptosis in H9c2 cells [21]. Tournefolic acid B (TAB, **126**) from *C. chinense* ameliorated hemodynamic parameters in isolated rat hearts and inhibited cardiomyocyte apoptosis. Conversely, TAB prevented myocardial I/R injury by inhibiting PI3K/AKT-mediated endoplasmic reticulum stress, oxidative stress, and apoptosis [27].

In addition, constituents of the genus *Clinopodium* also play an important role in the repair and functional regulation of the vascular system. Flavonoids and polyphenols, such as luteolin (**80**), naringenin (**93**), eriodictyol (**94**), ethyl (2R)-3-(3, 4-dihydroxyphenyl)-2-hydroxypropanate (**110**), caffeic acid (**113**), ethyl rosmarinate (**116**), and clinopodic acid B (**117**) from *C. chinense*, were involved in vascular endothelial-protective effects and significantly ameliorated high glucose-induced injury of HUVECs with EC_50_ values of approximately 3–36 μM [25]. Ethanolic extract of *C. tomentosum* improved the migration ability and angiogenic capacity of aortic endothelial cell pAEC, significantly increased the expression of FLK-1 mRNA, and ameliorated LPS-induced cell injury with angiogenic capacity [85]. An aqueous extract of *C. vulgare* increased CAT, GSH-Px, and SOD activities in the liver, kidneys, and brain. It also maintained the depletion of reduced glutathione GSH, and slightly decreased systolic blood pressure [86].

### 3.3. Anti-Inflammatory Activity

The anti-inflammatory effects of *C. chinense* were characterized by multi-target regulation, involving the inhibition of key signaling pathways and regulation of metabolic homeostasis. Ethyl acetate extract of *C. chinense* attenuated PA-induced inflammation through a dual pathway, exerting vasculo-protective effects. On the one hand, it inhibited TLR4 expression in HUVECs and blocked the downstream MyD88/TRIF/TRAF6 signaling pathway. This inhibited the phosphorylation of IκB kinase *β*, NF-κB and the MAPK family (JNK/ERK/p38), and reduced the release of inflammatory factors such as TNF-*α*, IL-1*β*, and IL-6. On the other hand, restoring eNOS activity by modulating the phosphorylation pattern of IRS-1 stimulated insulin-mediated NO production in PA-treated HUVECs and ameliorated impaired insulin signaling in the vascular endothelium [87]. This multi-pathway synergistic effect was further validated by evaluating the anti-inflammatory activity of camphor, menthol, and their equimolar combination. Camphor and menthol, the major compounds in *C. nepeta* EO, showed that the combination was more effective in inhibiting 5-lipoxygenase (72.5% vs. 48.3% for camphor and 52.9% for menthol), as well as COX-1 and COX-2 cyclooxygenases (78.1% and 79.4%, respectively, vs. 60.4% and 62.7% for camphor, 64.2% and 66.3% for menthol)[109].

Network pharmacological predictions, combined with experimental confirmation, show that the genus inhibits macrophage inflammation via the TLR4-NF-κB-iNOS/COX-2 axis. It also modulates key metabolic pathways such as arachidonic acid metabolism, histidine metabolism, alanine, aspartate and glutamate metabolism, and the pentose phosphate pathway. This results in reduced MDA levels and decreased pro-inflammatory factors, such as IL-6 and TNF-*α* in colon tissues of the UC model. In conclusion, *C. chinense* extract may alleviate ulcerative colitis by reducing systemic inflammation and modulating metabolism [88]. Caffeic acid, chlorogenic acid, and catechin from *C. vulgare* were proven to inhibit zymosan-induced COX-2 expression in bone marrow neutrophils [110]. Notably, specific targets in aqueous extracts of different *C. vulgare* species selectively inhibited p38/JNK MAPKs phosphorylation and MMP-9 activation, but had a weak effect on the COX-2/PGE2 pathway, whereas *C. bolivianum* inhibited the adhesion, invasion and biofilm formation of uroepithelial pathogenic *E. coli* by upregulating caveolin-1 [90]. The novel diterpene compounds, imbricatusol I (**66**) from *C. polycephalum* and buddlejasaponin IV (**48)** from *C. chinense*, were found to inhibit NO production in LPS-induced RAW 264.7 cells without affecting cell viability. These findings provide a new direction for the development of anti-inflammatory drugs targeting extracellular matrix protection [17,22].

The anti-inflammatory benefits of aqueous/alcoholic extracts of *C. gracile* are also demonstrated by a multimodal mechanism of action that is effective against acute neurogenic pain, inflammatory pain, and acute inflammation. These extracts can simultaneously decrease the levels of mediators, such as NO, MDA, PGE2, etc., in brain tissue and peripheral blood, and reduce intracellular ROS to baseline levels by inhibiting xanthine oxidase activity. This mechanism is associated with the suppression of the brain’s expression of NO, MDA, and PGE2, as well as the expression of NO, MDA, PGE2, IL-6, and TNF-*α* in peripheral blood [89]. Additionally, *C. nepeta* EO also exhibited strong anti-inflammatory properties against carrageenan-induced paw edema, with an IC_50_ value of 17.23 ± 0.32 μg·mL^−1^ compared to diclofenac [56].

### 3.4. Antimicrobial Activity

The genus *Clinopodium* exhibited broad-spectrum antimicrobial activity. The antimicrobial activity of EOs from more than 12 species of the genus *Clinopodium* has become a hot topic. In the area of antimicrobial resistance, *C. nepeta* EO demonstrated unique advantages against multi-drug resistant strains. For ampicillin-/ciprofloxacin-/gentamicin-resistant *Escherichia coli* JM109, its minimal inhibitory concentrations (MICs) were as low as 0.300–0.966 μL·mL^−1^ and the ratio of bactericidal concentration (MBC) to MIC was ≤2, confirming its potent bactericidal properties [91]. Notably, *C. nepeta* EO showed the highest activity against *Salmonella typhimurium* at 1250 µg·mL^−1^. The EO was more effective against *B. cereus* (2500 μg·mL^−1^) and *S. sanguinis* (2500 µg·mL^−1^). However, it was least active against *E. coli* and *Pseudomonas aeruginosa* [62]. An analysis of chemical fractions reveals conformational relationships: the synergistic effect of menthone (44.8%) and isomenthone (5.8%) from *C. candidissimum* EO resulted in a low MIC at 0.145 g·L^−1^ against phytopathogenic bacteria (e.g., *Pseudomonas lilacis*) and multi-targeting effects through the competitive inhibition of acetylcholinesterase (IC_50_ = 0.17–0.43 g·mL^−1^) [54]. *Calamintha nepeta* EO exhibited minimum inhibitory concentrations and bactericidal/fungicidal concentrations ranging from 0.937 to 3.75 µL·mL^−1^ and 0.937 to 15 µL·mL^−1^, respectively [56]. *Satureja calamintha nepeta* exhibits antifungal potency indicating that the methanolic extract is more active (>69.04% ± 2.06) than the aqueous extract (>29.76% ± 2.06) against all tested molds [111]. Furthermore, its EO shows strong activity against *E. coli* and *E. vekanda* strains, with MIC values of approximately 2.80 µg·mL^−1^ [35].

Regarding the potential for topical application, *C. sericeum* EO with *β*-stilbene (13.8%) and mangosteen (11.2%) as main constituents showed antimicrobial activity against Gram-negative and Gram-positive bacterial strains with MIC of 50–200 μg·mL^−1^ [69]. *C. chinense* extract also exhibited good inhibitory effects against *Escherichia coli*, *Staphylococcus aureus*, and *Proteus vulgaris* with MICs of 0.0625 g·mL^−1^; however, it showed no inhibition against *Sarcina lutea* and *Aspergillus niger*. This suggests that its antimicrobial spectrum is selective [112].

This selectivity was further elucidated in *C. menthifolium*. EO from the Babouch region showed the strongest antifungal activity against *Aspergillus terreuss*, *Microsporum canis*, and *Candida albicans* (MIC = 40–400 μg·mL^−1^), while EO from the Tabarka region showed a 42.5% repellency rate against the storage pest *Tribolium confusum*. These results indicate that geographical origin significantly influences biological activity by modulating terpene constituents [55]. EO from *Calamintha menthifolia* had strong antibacterial properties with *Staphylococcus albicans*, *Escherichia coli*, and *P. fluorescens* being the most susceptible strains and *P. vulgaris* being the least susceptible. However, it was less active than ciprofloxacin and *L. angustifolia* EO [113].

*C. brevicaly* EO showed an MIC of 125 μg·mL^−1^ against *Trichophyton rubrum*. Its antifungal activity was mechanistically related to the transmembrane permeability of terpene constituents [44]. The caryophyllene content of *C. brownei* EO has been reported to be positively correlated with the antibacterial activity. The bacterial growth inhibitory concentration was 3.11 mg·mL^−1^ for *Candida albicans* ATCC 10231, significantly lower than that of the positive control. This suggests that the antifungal activity is primarily due to the terpene constituents [45].

Besides the genus *Clinopodium* EOs, polyphenolic compounds in extracts are active antifungal constituents. *C. nubigenum* extracts obtained by supercritical fluid extraction contain thymol, carvacrol, (±)-6-hydroxy-2,5,7,8-tetramethylchroman-2-carboxylic acid, 5-hydroxyflavone, and icarrin. These extracts exhibit the most potent antifungal activity against *Colletotrichum musae*, *Mucor racemosus*, and *Fusarium verticillioides* with MICs of 400, 400, and 825 mg·L^−1^, respectively [114]. AcOEt extract of *C. nepeta* inhibited the swarming motility of *Pseudomonas aeruginosa* PA01 with an MIC of 12.0 ± 0.5 mm, showed the best activity of the violacein group sensing 35.42 ± 1.00% at 100 μg·mL^−1^, and disrupted bacterial biofilm formation [92]. Furthermore, *C. bolivianum* polysaccharides have more powerful activity against HIV-1 infection, with an IC_50_ of 4.72 µg·mL^−1^ [115]. The low polar fraction of *Calamintha baborensis* showed good inhibition against *staphylococci* and *E. coli*, with inhibition zone diameters of 19 mm and 19.2 mm, and MIC values of about 43 and 43.4 μg·mL^−1^, respectively. The main active compound against *staphylococci* was phenyl-*β*-D-glucuronide rhamnoside [46,116].

In addition to the different effects of species type and extraction method, the origin of the species also affects antimicrobial activity. For example, hexane extracts of *Calamintha sylvatica* from natural and in vitro propagated plantlets showed activity only against *Staphylococcus aureus* with MIC values at 6.25 and 3.33 m·mL^−1^, respectively. This activity may be correlated with the content of rosmarinic acid [117]. *S. calamintha* EO showed the best activity against *E. coli* K12 compared to the antibiotic streptomycin sulfate. Similarly, wild and domesticated *S. calamintha* showed differences with inhibitory diameters of 25.67 ± 0.58 mm and 48.67 ± 1.15 mm and MIC values of 1.49 ± 0.00 μg·mL^−1^ and 0.373 ± 0.00 μg·mL^−1^, respectively [97]. The combined action of Lactobacillus (LB) exopolysaccharides and *Satureja calamintha* extracts reduced the aggregation ability of enteropathogenic *Escherichia coli* and decreased the rate of their adhesion [118]. *C. vardarensis* EO exhibits selective antimicrobial activity against *Staphylococcus aureus* (MIC 21.25 µg·mL^−1^). The overall effect of EO–antibiotic combinations varied from synergistic (FICI ≤ 0.5) to antagonistic (FICI ≥ 2) depending on the bacterial strain tested [30]. Thus, this genus is a diverse source of candidates for developing novel antimicrobial agents through the multimodal action of terpenoid, phenolic acid and glycoside components.

### 3.5. Antitumor Activity

The antitumor activity of *C. chinense* is closely related to the structural diversity of its specific secondary metabolites. Compound **45**, a triterpenoid saponin with C-11 carbonyl/C-3 glucose modification from *C. chinense*, exhibited comparable cytotoxicity to the positive control, 10-hydroxycamptothecin, against mouse mammary adenocarcinoma 4T1 cells (IC_50_ = 7.4 μM and 7.6 μM, respectively) [8]. However, other terpenes from *C. chinense* showed no direct cytotoxicity (IC_50_ > 100 μM) against A549 and HepG2 cancer cell lines, which may be due to the lack of a C-11 carbonyl skeleton in the compounds or to the selectivity of the anticancer activity of the genus *Clinopodium* [119]. Nevertheless, compounds **28**–**30** and **32** significantly improved the insulin resistance index in HepG2 cells (42.7 ± 3.5% reduction in HOMA-IR), suggesting that they may indirectly affect the tumor microenvironment through metabolic regulatory pathways [13].

Similarly, petroleum ether, chloroform, and methanol extracts of *C. umbraculum* showed cytotoxic activity against HN-5 oral cancer cells with IC_50_ values of >250, >167, and 239.5 μg·mL^−1^, respectively. Buddlejasaponin IVa (**55**) and buddlejasaponin IV (**48)** isolated from *C. umbraculum* significantly increased the Bax/Bcl-2 ratio and activated caspase-9, inhibited the migration of HN-5 cells, and enhanced the cytotoxicity of compound **48** (IC_50_ = 58.3 μM) compared to the crude extract (IC_50_ > 167 μg·mL^−1^), indicating that the two saponins exerted their anticancer activities via the mitochondrial apoptotic pathway [94]. Compound **48** also showed considerable anticancer activity against A549, HCT116, MDA-MB-231, SNU638, and SK-HeP-1 cells [17].

In addition to triterpenoid saponins, EOs from the genus *Clinopodium* are also an important class of tumor-active constituents. The toxicity of *C. fenzlii* EO on HeLa cells showed concentration-dependent death. *C. fenzlii* VO at 1.4 and 0.7 mg·mL^−1^ significantly induced cell cytotoxicity with mortality rates of approximately 50% and 40%, respectively, while concentrations ranging from 2.9 to 23.22 mg·mL^−1^ significantly induced more than 70% of the cells to die [51]. *C. sericeum* EO (*β*-germacrene D 15%) exerted selective cytotoxic effects through non-oxidative stress pathways on T24 bladder cancer (IC_50_ = 0.19 mg·mL^−1^) and MCF-7 breast cancer (IC_50_ = 0.21 mg·mL^−1^) cells. Its constituents were also shown to exert cytotoxicity through the mitochondrial apoptotic pathway; they produced selective cytotoxicity, and the toxicity threshold for normal HEK-293 cells was high (IC_50_ = 0.38 mg·mL^−1^) [69]. *C. nepeta* EO showed anticancer activity against MCF-7, MDA-MB-231, and MDA-MB-436 breast cancer lines with IC_50_ values of 27.6, 31.2, and 36.5 µg·mL^−1^, respectively, demonstrating remarkable selectivity for cancer cells [109]. *Calamintha nepeta* EO showed cytotoxic activity against non-small cell lung cancer (A549) cells with IC_50_ values of 442.9 μg·mL^−1^ and 133.9 μg·mL^−1^ at 24 h and 48 h, respectively [120].

### 3.6. Antioxidant Activity

The antioxidant activity of the genus *Clinopodium* is significantly dependent on the extraction solvent. Aqueous extracts and organic solvent extracts (e.g., different concentrations of ethanol and methanol) showed significant differences in antioxidant capacity [95,98,101]. The *n*-BuOH extract of *Calamintha baborensis* was superior to the EtOAC extract in ABTS (81.7% inhibition), DPPH (80.99%), and FRAP (19.52 μM/mL), suggesting that high-polarity solvents are more conducive to the solubilization of its antioxidant components [46]. Similarly, the ethanolic extracts of *C. serpyllifolium* flower excelled in DPPH radical scavenging (92.14%) and FRAP reducing power (3.138 ± 0.08 mg TE/g). However, its aqueous extract was less active in ABTS and CUPRAC tests, which may be related to the extraction efficiency of polar solvents for phenolic compounds [7]. The methanolic extract of *C. vulgare* demonstrated broad-spectrum antioxidant activity, with CUPRAC and FRAP values of 44.32 and 87.25 mg TE/g, respectively. The CV3 fractions, which were separated by reversed-phase chromatography, showed an extremely strong free radical scavenging activity, with an IC_50_ value of 0.02 mg·mL^−1^ in the DPPH test and 0.0002 mg·mL^−1^ in the ABTS test. This suggests that methanol is a more suitable solvent for extracting highly active polyphenols [99,100].

In addition, the polyphenol content and antioxidant strength are influenced by the growing environment, collection time, extraction method, and cultivation method [77,121]. The EOs of wild *Satureja calamintha* (EOSS) showed higher activity in both the DPPH and FRAP assays than that of the cultivated variety (EOSD), possibly due to variations in the accumulation of secondary metabolites [97]. *C. bolivianum* extracts produced using various extraction methods revealed the technical benefits of ultrasound-assisted extraction. This method increased the total phenolic content by up to 182.2 mg GAE/g and the antioxidant capacity by up to 1470.0 µmol TE/g, which is an 83% increase in efficiency compared to the traditional methods [95]. Notably, the species-specific difference is reflected in the fact that the butanol extract of *C. nepeta* exceeded the *α*-tocopherol activity twofold in the FRAP test (A_0.5_ = 17.42 vs. 34.93 µg·mL^−1^) [92]. Furthermore, *C. nepeta* EO revealed a synergistic effect when combined equimolarly, achieving IC_50_ values of 10.3 µg·mL^−1^ in the DPPH test and 8.9 µg·mL^−1^ in the ABTS test. This surpasses the efficacy of ascorbic acid with an IC_50_ of 12.4 µg·mL^−1^ [109]. In contrast, EOs from *C. sericeum* and *C. brownie* showed very low antioxidant activity [45,52,69]. These findings systematically reveal the potential of *C. nepeta* as a natural antioxidant for the development of the genus *Clinopodium*, while suggesting the key role of germplasm resource screening and extraction process optimization for acquiring its active components [122].

The total phenolic content was shown to be significantly correlated with DPPH (r = −0.974), ABTS (r = −0.944), and FRAP (r = 0.957) activities. For example, *C. vulgare* aqueous extracts exhibited strong radical scavenging due to the high polyphenol content (174.42 mg GAE/g) [123]. Flavonoids such as quercetin (**104**) and rutin were found in *C. serpyllifolium* flower extracts at a high concentration of 214.03 mg QE/g, which was directly correlated with its DPPH activity [98]. The enrichment characteristics of rosemarinic acid (34.21 mg·g^−1^) and ellagic acid (29.31 mg·g^−1^), as well as protocatechuic acid and chlorogenic acid, are of particular interest, as they may be the key material basis for its significant lipid antioxidant protection [99,101]. In conclusion, the antioxidant capacity of the genus *Clinopodium* is dominated by polyphenols and flavonoids, and the characteristic compounds (e.g., rosemarinic acid) play a key role.

### 3.7. Insecticidal Effect

EOs from the genus *Clinopodium* exhibit significant insecticidal activity. *C. nubigenum* EO demonstrated remarkably inhibited oviposition of *Lavandula sericata* at 0.8 μL·cm^–2^ for 3 h, and oviposition inhibition of *C. nubigenum* EO was 89.5% after 24 h, better than that of *Lavandula angustifolia* Mill, a well-known medicinal plant. *C. nubigenum* EO showed potent toxicity with an LC_50_ value of 0.07 μL·cm^–2^ against the eggs of *L. sericata* by contact/fumigation, and LD_50_ value of 0.278 μL per individual by topical application on the adults. It is suggested that regarding *C. nubigenum* EO on acetylcholine esterase of *L. sericata*, with its IC_50_ value of 67.450 mg·L^–1^, the toxicity target of *C. nubigenum* EO is neural sites [40].

EO from *C. chinense* displayed both fumigant toxicity (LC_50_ = 423.39 μg·L^−1^ against *Liposcelis bostrychophila*) and contact toxicity (LC₅₀ = 215.25 μg·cm^−2^). Its major components, bornyl acetate and piperitone, showed enhanced activity with fumigant LC₅₀ values of 351.69 and 311.12 μg·L^−1^, respectively, and contact LC₅₀ values of 321.42 and 139.74 μg·cm^−2^, respectively [48]. *C. menthifolium* EO showed moderate contact toxicity against *Tribolium confusum*, resulting in 27.5–32.5% mortality at a 5% concentration [55].

Acaricidal activity was observed for *C. nepeta* (52%) and *C. sylvatica* (60%) at 2 mg·mL^−1^, with no apparent toxicity to honeybees [39]. Cultivation methods influenced chemical composition, increasing menthol content while decreasing pulegone content. Wild and cultivated plant oils exhibited strong insecticidal activity against stored-product pests (*Tribolium confusum*, *Rhyzopertha dominica*, *Sitophilus oryzae*), with LD₅₀ values of 0.004–0.011 μL·cm^−2^ (contact) and 1.988–10.817 μL·L^−1^ (fumigation) [38]. Notably, *S. calamintha* EO showed sex-dependent toxicity against *Callosobruchus maculatus* (100% male mortality vs. 86.66% female mortality at LC₅₀ = 2.17 μL·L^−1^) and strong repellent activity (91.67% repellency) [49]. Therefore, EOs of the genus *Clinopodium* have the potential to be developed as natural insecticides or fumigants for controlling insects in stored grains.

### 3.8. Others

In addition to the above mentioned compounds, luteolin (**80**), eriodictyol (**94**), ethyl rosmarinate (**116**), and clinopodic acid B (**117**), which are found in *C. chinense*, are potential lead compounds for antidiabetic drugs due to their *α*-glucosidase inhibitory activity with IC_50_ values ranging from 0.6 to 2.0 μM [25]. Moreover, aqueous extracts of *Calamintha officinalis* exhibited antidiabetic and hypolipidemic effects, indicating that these activities are primarily mediated by hydrophilic constituents [124]. *C. vulgare* extracts alleviated the scopolamine-induced downregulation of p-CREB/BDNF signaling, exhibit recognition memory retention and acetylcholinesterase inhibitory activity [125]. Beyond medicinal applications, EO from *Calamintha* leaves has also displayed anticorrosive potential for protecting mild steel, highlighting its industrial relevance [126].

## 4. Quality Evaluation

The Chinese Pharmacopoeia has always used buddlejasaponin IVb (**21**) as a reference compound. It was first developed in chloroform–methanol–glacial acetic acid–water (7:2.5:1:0.5), then developed with 10% sulfuric acid ethanol solution, and inspected under sunlight and ultraviolet light with a wavelength of 365 nm. However, the Pharmacopoeia still lacks method to determine the content and evaluate the quality of *C. chinense*. Quality control of the genus *Clinopodium* reported in the references is mainly for *C. chinense* and *C. gracile*, with the active components, flavonoids and saponins, as the predominant quantitative criteria. The HPLC characteristic spectrum of *C. chinense* was established with the following reference compounds: buddleoside (**81**), didymin (**96**), naringenin (**93**), apigenin (**79**), isosakuranetin (**95**), acacetin (**91**), and buddlejasaponin IVb (**21**). The contents of **95** and **96** (wavelength at 290 nm) and **81** (wavelength at 330 nm) were 4.19–12.14, 1.65–2.87, and 0.90–5.93 mg·g^–1^, respectively [127]. Qi et al. established a fingerprint spectrum of *C. chinense* extract using UPLC-Q-TOF-MS and identified 25 common peaks. They identified seven major components with anti-AUB activity, including compounds **21**, **79**, **93**, hesperidin (**97**), kaempferol (**103**), quercetin (**104**), and saikosaponin a (**46**) [71].

In the thin-layer chromatography of *C. gracile*, a spot of the same color appeared at the corresponding position in the chromatogram of saikosaponin a (**46**) as the reference compound. The average moisture content of *C. gracile* was determined to be 10.10%, the total ash content to be 9.73%, the acid-insoluble ash content to be 1.06%, and the dilute ethanol extract to be 23.54%. The average rosmarinic acid content was determined to be 0.56% [128].

## 5. Toxicity

The acute and sub-acute toxicity of *C. vulgare* lyophilized aqueous extract (CVE) was demonstrated by intraperitoneal injection (i.p.), resulting in death due to difficulty breathing in mice and rats. The LD_50_ values were 675 mg·kg^–1^ (mice) and 500 mg·kg^–1^ (rats), respectively. These results indicate that acute intraperitoneal injection resulted in central nervous system toxic effects. In contrast, the LD_50_ for oral administration was higher than 2000 mg·kg^–1^ in both mice and rats. Furthermore, oral administration of CVE did not produce any toxic effects on hematology, blood and urine biochemistry, or histomorphometry of the pancreas, liver, spleen, or kidneys. This indicates that the flavonoids in the meso-extract, caffeic acid oligomers, saponins, and rosemarinic acid, which are the major compounds in the meso-extract, did not cause any hematological, biochemical, or histopathological changes [75]. Oral toxicity tests also showed that the LD_50_ of *C. nepeta* EO was 2500 mg/kg in Wistar rats [56] and 1500 mg/kg in Swiss mice [58].

Similarly, the total flavonoids in *C. chinense*, as the major active constituents, did not cause toxicity or death at the maximum oral doses of 4000 mg/kg in rats and 5000 mg/kg in mice, respectively. Moreover, no changes in food intake, water intake, body weight, chemical and hematological parameters, organ weights, gross pathology or histopathology were observed in rats fed continuously by gavage for four weeks [129]. The toxicity experiments provide a practical guide for selecting safe doses of *C. vulgare*, *C. nepeta*, and *C. chinense* for further studies in animal research or clinical trials.

## 6. Pharmacokinetics

As a traditional Chinese herbal medicine, *C. chinense* has been used to treat various gynecological bleeding disorders. However, the in vivo metabolism of its complex chemical components still needs to be analyzed in depth. Li et al. developed an LC-MS/MS method that simultaneously quantified five components in rat plasma, including buddlejasaponin IVb (**21**), saikosaponin a (**46**), apigenin (**79**), acacetin 7-*O*-glucuronide (**87)**, and didymin (**96**). The lowest limits of quantification (LLOQs) were 5.3, 10.5, 1.85, 1, and 4.7 ng·mL^–1^, respectively. This method is simple, rapid, and stable. It also lays a methodological foundation for the systematic development of multi-component pharmacokinetic studies of *C. chinense* extracts [130]. It is worth noting that the existing studies have only monitored specific polar components, while the metabolic profiles of possible nonpolar-soluble components (e.g., sterols and volatile oils) and bound components of *C. chinense* remain unknown. This may lead to a biased perception of its overall pharmacokinetic behavior.

Changes in the expression and activity of drug metabolizing enzymes (DMEs) are key factors affecting the disposition of herbal components in vivo. A recent study showed that the ethanolic extract of *Calamintha incana*, a herb commonly used in the Middle East, significantly increased the hepatic level of the CYP3A11 gene—more than 10-fold—in mice after one month of low-dose intervention. Neither high nor low doses induced hepatic histopathological damage [131]. These results suggest that *C. incana* may mildly activate the CYP3A subfamily of enzymes via specific constituents, thereby affecting the metabolic clearance rate of its own or co-administered drugs. In addition, the expression of other CYP isoforms, such as CYP2C29, CYP2D9, and CYP1A1, did not change significantly, suggesting that *C. incana* selectively regulates metabolizing enzymes, which is consistent with the action characteristics of traditional Chinese medicine: “multi-component—precise target”.

## 7. Conclusions and Perspectives

This paper summarizes the latest findings on the chemical composition, pharmacological activity, quality evaluation, toxicity, and pharmacokinetics of the genus *Clinopodium* over the past decade. The active constituents include terpenoids and their saponins, flavonoids, and phenylpropanoids. Of these, 78 triterpenoids were identified, 36 of which were new. In addition, numerous studies on the chemical constituents of EOs and their antioxidant and antimicrobial activities have been reported. These findings suggest that terpenoids and EOs from different species types are two hot topics in the research of the genus *Clinopodium*. So far, most studies have focused on the important role of the genus *Clinopodium* in hemostasis, cardiovascular and myocardial cell protection, and anti-inflammatory analgesia, as well as on its antimicrobial, antitumor, and antioxidant properties; however, the mechanism of their biological activity has not been fully elucidated. The EOs of the genus *Clinopodium* also have fumigation toxicity against insects. However, there are no references proving their toxicity and side effects on humans. Thus, further research is needed to evaluate these effects. In addition, although the flavonoids of the genus *Clinopodium* have been reported in pharmacokinetic studies, pharmacokinetic and pharmacodynamic analyses of other components such as terpenoids need to be further understood. The various functions of the bioactive compounds of the genus *Clinopodium* are crucial for its drug development, especially for the application of hemostatic drugs.

## Figures and Tables

**Table 1:** Compounds from the genus *Clinopodium* L.

No.	Name	Sources	Organs	Extraction Solvent	Collection Areas	Ref.
Triterpenoids						
**1** *^, 1^	clinopodiside VIII	*C. chinense*	Aerial parts	70% ethanol	Bozhou, Anhui Province, China	[8]
**2** *	clinopodiside IX	*C. chinense*	Aerial parts	70% ethanol	Bozhou, Anhui Province, China	[8]
**3** *	clinopodiside X	*C. chinense*	Aerial parts	70% ethanol	Bozhou, Anhui Province, China	[8]
**4** *	clinopodiside XI	*C. chinense*	Aerial parts	70% ethanol	Bozhou, Anhui Province, China	[8]
**5**	16*β*,21*β*,23,28-tetrahydroxyoleana-9(11),12(13)-diene-3-yl-[*β*-D-glucopyranosyl-(1→2)]-[*β*-D-glucopyranosyl-(1→3)]-*β*-D-fucopyranoside	*C. chinense*	Aerial parts	70% ethanol	Bozhou, Anhui Province, China	[8]
**6** *	clinopodiside VI	*C. polycephalum*	Aerial parts	70% aqueous EtOH	Anhui Province, China	[9]
**7** *	clinopodiside VII	*C. chinense*	Aerial parts	70% ethanol	Bozhou, Anhui Province, China	[8]
**8**	saikosaponin g	*C. chinense*	Aerial parts	70% ethanol	Bozhou, Anhui Province, China	[8]
**9**	pleurosaponin I	*C. gracile*	Whole herb	70% aqueous EtOH	Shaanxi province, China	[10]
**10**	16*β*,23,28-trihydroxyoleana-9(11),12(13)-dien-3-yl-[*β*-D-glucopyranosyl-(1→2)]-[*β*-D-glucopyranosyl (1→3)]-*β*-D-fucopyranoside	*C. chinense*	Aerial parts	70% ethanol	Bozhou, Anhui Province, China	[8]
**11** *	clinopodiside XII	*C. chinense*	Aerial parts	70% ethanol	Bozhou, Anhui Province, China	[8]
**12**	16*β*,23,28-trihydroxyoleana-9(11),12(13)-diene-3-yl-[*β*-D-glucopyranosyl-(1→4)-*β*-D-glucopyranosyl-(1→6)-*β*-D-glucopyranosyl-(1→3)]-[*β*-D-glucopyranosyl-(1→2)]-*β*-D-fucopyranoside	*C. chinense*	Aerial parts	70% ethanol	Bozhou, Anhui Province, China	[8]
**13**	saikogenin A	*C. chinense*	Aerial parts	80% EtOH	Putian, Fujian province, China	[11]
**14**	prosaikogenin A	*C. chinense*	Aerial parts	80% EtOH	Putian, Fujian province, China	[11]
**15** *	clinoposaponin F	*C. chinense*	Aerial parts	70% ethanol	Bozhou, Anhui province, China	[12]
**16** *	clinoposaponin G	*C. chinense*	Aerial parts	70% ethanol	Bozhou, Anhui province, China	[12]
**17** *	clinoposaponin E	*C. chinense*	Aerial parts	70% EtOH	Bozhou, Anhui Province, China	[13]
**18** *	saikosaponin c	*C. polycephalum*	Aerial parts	70% aqueous EtOH	Anhui Province, China	[9]
		*C. gracile*	Whole herb	70% aqueous EtOH	Shaanxi province, China	[10]
**19**	clinopodiside I	*C. polycephalum*	Aerial parts	70% aqueous EtOH	Anhui Province, China	[9]
**20**	saikosaponin b1	*C. polycephalum*	Aerial parts	70% aqueous EtOH	Anhui Province, China	[9]
**21**	buddlejasaponin IVb	*C. polycephalum*	Aerial parts	70% aqueous EtOH	Anhui Province, China	[9]
		*C. gracile*	Whole herb	70% aqueous EtOH	Shaanxi province, China	[10]
		*C. chinense*	Aerial parts	80% EtOH	Putian, Fujian province, China	[11]
**22** *	clinograsaponin B	*C. gracile*	Whole herb	70% aqueous EtOH	Shaanxi province, China	[10]
**23**	tibesaikosaponin IV	*C. gracile*	Whole herb	70% aqueous EtOH	Shaanxi province, China	[10]
**24** *	clinoposaponin D	*C. gracile*	Whole herb	70% aqueous EtOH	Shaanxi province, China	[10]
		*C. chinense*	Aerial parts	80% EtOH	Putian, Fujian province, China	[11]
**25**	11*α*,16*β*,23,28-tetrahydroxyolean-12-en-3*β*-yl-[*β*-D-glucopyranosyl-(1→2)]-[*β*-D-glucopyranosyl-(1→3)]-*β*-D-fucopyranoside	*C. chinense*	Aerial parts	80% EtOH	Putian, Fujian province, China	[11]
**26** *	clinopoditerpene B	*C. chinense*	Aerial parts	70% ethanol	Bozhou, Anhui Province, China	[14]
**27** *	clinopoditerpene C	*C. chinense*	Aerial parts	70% ethanol	Bozhou, Anhui Province, China	[14]
**28** *	clinopoditerpene D	*C. chinense*	Aerial parts	70% EtOH	Bozhou, Anhui Province, China	[13]
**29**	perovskiaditerpenoside B	*C. chinense*	Aerial parts	70% EtOH	Bozhou, Anhui Province, China	[13]
**30**	3*β*-hydroxy-12-*O*-*β*-D-glucopyranosyl-8,11,13-abietatrien-7-one	*C. chinense*	Aerial parts	70% EtOH	Bozhou, Anhui Province, China	[13]
**31**	12-*O*-*β*-D-glucopyranosyl-3,11,16-trihydroxyabieta-8,11,13-triene	*C. chinense*	Aerial parts	70% EtOH	Bozhou, Anhui Province, China	[13]
**32**	cussoracoside A	*C. chinense*	Aerial parts	70% EtOH	Bozhou, Anhui Province, China	[13]
**33**	15-hydroxy-12-oxo-abietic acid	*C. bolivianum*	Aerial parts	Aqueous extract	Ingavi province, La Paz department, Bolivia	[15]
**34**	12*α*-hydroxy-abietic acid	*C. bolivianum*	Aerial parts	Aqueous extract	Ingavi province, La Paz department, Bolivia	[15]
**35**	(−)-jolkinolide E	*C. bolivianum*	Aerial parts	Aqueous extract	Ingavi province, La Paz department, Bolivia	[15]
**36**	15-hydroxy-dehydroabietic acid	*C. bolivianum*	Aerial parts	Aqueous extract	Ingavi province, La Paz department, Bolivia	[15]
**37**	clinopodiside G	*C. chinense*	Aerial parts	70% EtOH	Bozhou, Anhui Province, China	[13]
**38** *	clinopodiside H	*C. chinense*	Aerial parts	70% EtOH	Bozhou, Anhui Province, China	[16]
**39**	clinopodiside D	*C. chinense*	Aerial parts	80% EtOH	Putian, Fujian province, China	[11]
**40**	clinoposaponin XVI	*C. chinense*	Aerial parts	70% ethanol	Bozhou, Anhui Province, China	[8]
**41**	clinoposaponin XX	*C. chinense*	Aerial parts	70% ethanol	Bozhou, Anhui Province, China	[8]
**42**	clinoposaponin XIX	*C. chinense*	Aerial parts	70% ethanol	Bozhou, Anhui Province, China	[8]
**43**	clinoposaponin XI	*C. gracile*	Whole herb	70% aqueous EtOH	Shaanxi province, China	[10]
**44**	clinoposaponin IV	*C. gracile*	Whole herb	70% aqueous EtOH	Shaanxi province, China	[10]
**45**	saikogenin F	*C. chinense*	Aerial parts	80% EtOH	Putian, Fujian province, China	[11]
**46**	saikosaponin a	*C. chinense*	Aerial parts	70% ethanol	Bozhou, Anhui province, China	[12]
**47**	clinoposaponin XV	*C. gracile*	Whole herb	70% aqueous EtOH	Shaanxi province, China	[10]
		*C. chinense*	Whole grass	MeOH	Wonju, Gangwon, Korea	[17]
**48**	buddlejasaponin IV	*C. chinense*	Aerial parts	70% ethanol	Bozhou, Anhui Province, China	[8]
		*C. polycephalum*	Aerial parts	70% aqueous EtOH	Anhui Province, China	[9]
		*C. gracile*	Whole herb	70% aqueous EtOH	Shaanxi province, China	[10]
		*C. chinense*	Aerial parts	80% EtOH	Putian, Fujian province, China	[11]
		*C. chinense*	Whole grass	MeOH	Wonju, Gangwon, Korea	[17]
		*C. umbrosum*	Aerial parts	Extraction using methanol	Noshahr, Mazandaran, Iran	[18]
**49** *	clinopoursaponin A	*C. chinense*	Aerial parts	70% ethanol	Bozhou, Anhui Province, China	[8]
**50** *	clinopoursaponin B	*C. chinense*	Aerial parts	70% ethanol	Bozhou, Anhui Province, China	[8]
**51** *	clinopoursaponin C	*C. chinense*	Aerial parts	70% ethanol	Bozhou, Anhui Province, China	[8]
**52** *	clinopoursaponin D	*C. chinense*	Aerial parts	70% ethanol	Bozhou, Anhui Province, China	[8]
**53**	comastomasaponin E	*C. gracile*	Whole herb	70% aqueous EtOH	Shaanxi province, China	[10]
**54**	saikosaponin b_3_	*C. gracile*	Whole herb	70% aqueous EtOH	Shaanxi province, China	[10]
**55**	buddlejasaponin IVa	*C. gracile*	Whole herb	70% aqueous EtOH	Shaanxi province, China	[10]
		*C. chinense*	Aerial parts	80% EtOH	Putian, Fujian province, China	[11]
		*C. chinense*	Aerial parts	70% EtOH	Bozhou, Anhui Province, China	[13]
		*C. umbrosum*	Aerial parts	Extraction using methanol	Noshahr, Mazandaran, Iran	[18]
**56** *	Polycephalum A	*C. polycephalum*	Whole grass	70% ethanol	Liangwang mountain, Kunming, Yunnan, China	[19]
**57** *	clinograsaponin A	*C. gracile*	Whole herb	70% aqueous EtOH	Shaanxi province, China	[10]
**58** *	clinoposide A	*C. chinense*	Aerial parts	70% ethanol	Bozhou, Anhui Province, China	[20]
**59**	clinoposide B	*C. chinense*	Aerial parts	70% ethanol	Bozhou, Anhui Province, China	[20]
**60** *	clinoposide C	*C. chinense*	Aerial parts	70% ethanol	Bozhou, Anhui Province, China	[20]
**61** *	clinoposide D	*C. chinense*	Aerial parts	70% ethanol	Bozhou, Anhui Province, China	[20]
**62** *	clinoposide E	*C. chinense*	Aerial parts	70% ethanol	Bozhou, Anhui Province, China	[20]
**63** *	clinoposide F	*C. chinense*	Aerial parts	70% ethanol	Bozhou, Anhui Province, China	[20]
**64** *	clinoposide G	*C. chinense*	Aerial parts	70% ethanol	Bozhou, Anhui Province, China	[21]
**65** *	clinoposide H	*C. chinense*	Aerial parts	70% ethanol	Bozhou, Anhui Province, China	[21]
**66** *	imbricatusol I	*C. polycephalum*	Whole grass	70% ethanol	Kunming, Yunnan province, China	[22]
**67** *	saturol I	*C. polycephalum*	Whole grass	70% ethanol	Kunming, Yunnan province, China	[22]
**68** *	3*β*-22,25-dihydroxy-tirucalla-7,23-diene	*C. polycephalum*	Whole grass	70% ethanol	Kunming, Yunnan province, China	[22]
**69**	maslinic acid	*C. polycephalum*	Whole grass	70% ethanol	Kunming, Yunnan province, China	[22]
**70**	2*α*,3*α*-dihydroxyolean-12-en-28-oic acid	*C. polycephalum*	Whole grass	70% ethanol	Kunming, Yunnan province, China	[22]
**71**	hederagenin	*C. polycephalum*	Whole grass	70% ethanol	Kunming, Yunnan province, China	[22]
**72**	2*α*,3*α*-dihydroxyursolic acid	*C. polycephalum*	Whole grass	70% ethanol	Kunming, Yunnan province, China	[22]
**73**	alphitolic acid	*C. polycephalum*	Whole grass	70% ethanol	Kunming, Yunnan province, China	[22]
**74**	arjunglucoside I	*C. polycephalum*	Aerial parts	70% aqueous EtOH	Anhui Province, China	[9]
**75**	clinopodiside II	*C. polycephalum*	Aerial parts	70% aqueous EtOH	Anhui Province, China	[9]
**76** *	chinense A	*C. chinense*	Whole herb	70% EtOH	Guilin, Guangxi, China	[23]
**77** *	clinopoditerpene E	*C. chinense*	Whole herb	70% EtOH	Guilin, Guangxi, China	[23]
78 *	(4*S*,9*S*)-9-hydroxyjasmololone	*C. chinense*	Aerial parts	70% EtOH	Bozhou, Anhui province, China	[24]
Flavonoids						
**79**	apigenin	*C. chinense*	Whole plant	80% EtOH	Putian, Fujian province, China	[25]
			Aerial parts	70% ethanol	Anhui province, China	[26]
**80**	luteolin	*C. chinense*	Whole plant	80% EtOH	Putian, Fujian province, China	[25]
			Aerial parts	70% ethanol	Anhui province, China	[26]
**81**	buddleoside	*C. chinense*	Whole plant	80% EtOH	Putian, Fujian province, China	[25]
			Aerial parts	70% ethanol	Anhui province, China	[26]
**82**	apigenin-7-*O*-*β*-D-glucuronide	*C. chinense*	Aerial parts	70% ethanol	Anhui province, China	[26]
**83**	apigenin-7-*O*-*β*-D-glucuronopyranoside	*C. chinense*	Aerial parts	70% ethanol	Anhui province, China	[26]
**84**	apigenin 7-*O*-*β*-D-pyranglycuronate butyl ester	*C. chinense*	Aerial parts	70% ethanol	Anhui province, China	[26]
**85**	apigenin-7-*O*-*α*-L-rhamnopyranosyl (1→6)-*β*-D-glucopyranoside	*C. chinense*	Aerial parts	70% ethanol	Anhui province, China	[26]
**86**	luteolin-4′-*O*-*β*-D-glucopyranoside	*C. chinense*	Aerial parts	70% ethanol	Anhui province, China	[26]
**87**	luteolin-7-*O*-*β*-D-pyranglycuronate butyl ester	*C. chinense*	Aerial parts	70% ethanol	Anhui province, China	[26]
**88**	luteolin-7-O-*β*-D-glucuronide methyl ester	*C. chinense*	Aerial parts	70% EtOH	Bozhou, Anhui province, China	[24]
**89**	luteolin-7-*O*-rutinoside	*C. chinense*	Aerial parts	70% ethanol	Anhui province, China	[26]
**90**	luteolin-7-*O*-neohesperidoside	*C. chinense*	Aerial parts	70% ethanol	Anhui province, China	[26]
**91**	acacetin	*C. chinense*	Aerial parts	70% ethanol	Anhui province, China	[26]
**92**	acacetin 7-*O*-glucuronide	*C. chinense*	Aerial parts	70% ethanol	Anhui province, China	[26]
**93**	naringenin	*C. chinense*	Whole plant	80% EtOH	Putian, Fujian province, China	[25]
			Aerial parts	70% ethanol	Anhui province, China	[26]
**94**	eriodictyol	*C. chinense*	Whole plant	80% EtOH	Putian, Fujian province, China	[25]
**95**	isosakuranetin	*C. chinense*	Whole plant	80% EtOH	Putian, Fujian province, China	[25]
			Aerial parts	70% ethanol	Anhui province, China	[26]
**96**	didymin/neoponcirin	*C. chinense var. shibetchense* (H. Lev) Koidz	Whole grass	MeOH	Wonju, Gangwon, Korea	[17]
		*C. chinense*	Whole plant	80% EtOH	Putian, Fujian province, China	[25]
			Aerial parts	70% ethanol	Anhui province, China	[26]
**97**	hesperidin	*C. chinense*	Whole plant	80% EtOH	Putian, Fujian province, China	[25]
			Aerial parts	70% ethanol	Anhui province, China	[26]
**98**	prunin	*C. chinense*	Aerial parts	70% ethanol	Anhui province, China	[26]
**99**	naringenin 7-*O*-rutinoside/isonaringin	*C. chinense var. shibetchense* (H. Lev) Koidz	Whole grass	MeOH	Wonju, Gangwon, Korea	[17]
		*C. chinense*	Aerial parts	70% ethanol	Anhui province, China	[26]
**100**	naringenin-7-*O*-*β*-D-glucuronide	*C. chinense*	Aerial parts	70% EtOH	Bozhou, Anhui province, China	[24]
**101** *	polycephalum B	*C. polycephalum*	Whole grass	70% ethanol	Liangwang mountain, Kunming, Yunnan, China	[19]
**102**	isosakuranin	*C. chinense*	Aerial parts	70% ethanol	Anhui province, China	[26]
**103**	kaempferol	*C. chinense*	Aerial parts	70% ethanol	Anhui province, China	[26]
**104**	quercetin	*C. chinense*	Aerial parts	70% ethanol	Anhui province, China	[26]
**105**	kaempferol-3-*O*-glucorhamnoside	*C. chinense*	Aerial parts	70% ethanol	Anhui province, China	[26]
Phenylpropanoids						
**106**	(+)-isolariciresinol	*C. chinense*	Aerial parts	70% ethanol	Bozhou, Anhui province, China	[12]
**107**	fraxiresinol	*C. chinense*	Aerial parts	70% ethanol	Bozhou, Anhui province, China	[12]
**108**	8-hydroxy-7′-epipinoresinol	*C. chinense*	Aerial parts	70% ethanol	Bozhou, Anhui province, China	[12]
**109**	deltoignan A	*C. chinense*	Aerial parts	70% ethanol	Bozhou, Anhui province, China	[12]
**110**	ethyl (2*R*)-3-(3,4-dihydroxyphenyl)-2-hydroxypropanoate	*C. chinense*	Whole plant	80% EtOH	Putian, Fujian province, China	[25]
**111**	2-methoxy-4-(2-propenyl)-phenyl-*β*-D-glucopyranoside	*C. chinense*	Aerial parts	70% EtOH	Bozhou, Anhui province, China	[24]
**112**	ethyl (2*E*)-3-(3,4-dihydroxyphenyl) prop-2-enoate	*C. chinense*	Whole plant	80% EtOH	Putian, Fujian province, China	[25]
**113**	caffeic acid	*C. chinense*	Whole plant	80% EtOH	Putian, Fujian province, China	[25]
			Aerial parts	70% ethanol	Anhui province, China	[26]
**114**	ethyl (2*E*)-3-(2,3,4-trihydroxyphenyl) prop-2-enoate	*C. chinense*	Whole plant	80% EtOH	Putian, Fujian province, China	[25]
**115**	rosmarinic acid	*C. umbrosum*	Aerial parts	Methanol	Noshahr, Mazandaran, Iran	[18]
**116**	ethyl rosmarinate	*C. chinense*	Whole plant	80% EtOH	Putian, Fujian province, China	[25]
**117**	clinopodic acid B	*C. chinense*	Whole plant	80% EtOH	Putian, Fujian province, China	[25]
**118**	*p*-hydroxycinnamic acid	*C. chinense*	Aerial parts	70% ethanol	Anhui province, China	[26]
**119**	salicifoliol	*C. chinense*	Aerial parts	70% ethanol	Bozhou, Anhui Province, China	[12]
**120**	isofraxidin	*C. chinense*	Aerial parts	70% ethanol	Bozhou, Anhui province, China	[12]
Others						
**121**	*cis*-3-[2-[1-(3,4-dihydroxy-phenyl)-1-hydroxymethyl]-1,3-ben-zodioxol-5-yl]-(E)-2-propenoic acid	*C. chinense*	Aerial parts	70% ethanol	Anhui province, China	[26]
**122**	mesaconic acid	*C. chinense*	Aerial parts	70% ethanol	Anhui province, China	[26]
**123**	gentisic acid 5-*O*-*β*-D-(6′-salicylyl)-glucopyranoside	*C. chinense*	Aerial parts	70% ethanol	Anhui province, China	[26]
**124**	4-hydroxyl-3-methoxyphenyl-1-propane-1,2-diol	*C. chinense*	Aerial parts	70% ethanol	Bozhou, Anhui province, China	[12]
**125**	blumenol A	*C. chinense*	Aerial parts	70% ethanol	Bozhou, Anhui province, China	[12]
**126**	tournefolic acid B	*C. chinense*	/	/	/	[27]
**127** *	(*E*)-6-[9*R*-(*β*-D-glucopyranosyloxy) butylidene]-1,1,5-trimethyl-4-cyclohexen-3-one	*C. chinense*	Whole herb	70% EtOH	Guilin, Guangxi, China	[23]
**128**	(*E*)-6-[9*S*-(*β*-D-glucopyranosyloxy) butylidene]-1,1,5-trimethyl-4-cyclohexen-3-one	*C. chinense*	Whole herb	70% EtOH	Guilin, Guangxi, China	[23]
**129**	blumenol C 9-*O*-*β*-D-glucopyranoside	*C. chinense*	Whole herb	70% EtOH	Guilin, Guangxi, China	[23]
**130**	(6*R*,9*R*)-3-oxo-*α*-ionol-9-*O*-*β*-D-glucopyranoside	*C. chinense*	Whole herb	70% EtOH	Guilin, Guangxi, China	[23]
**131** *	chinense B	*C. chinense*	Whole herb	70% EtOH	Guilin, Guangxi, China	[23]
**132**	phaseic acid	*C. chinense*	Aerial parts	70% EtOH	Bozhou, Anhui province, China	[24]
**133**	*cyclo*-(*S*-Pro-*R*-Leu)	*C. chinense*	Aerial parts	70% EtOH	Bozhou, Anhui province, China	[24]
**134**	vomifoliol	*C. chinense*	Aerial parts	70% EtOH	Bozhou, Anhui province, China	[24]
**135**	*p*-mentha-3,8-dien-1,2-diol	*C. chinense*	Aerial parts	70% EtOH	Bozhou, Anhui province, China	[24]
**136**	corchoionol C	*C. chinense*	Aerial parts	70% EtOH	Bozhou, Anhui province, China	[24]

^1^ Compounds with * are new compounds.

**Table 3:** Pharmacological activity of the extract, fraction, or compounds from the genus *Clinopodium*.

Activity	Source	Extract, Fraction, or Compounds	Pharmacological Effects	Ref.
Hemostatic activity	*C. chinense*	Buddlejasaponin IVa (**55**), clinopodiside D (**39**), saikogenin A (**13**), saikogenin F (**45**), prosaikogenin A (**14**), buddlejasaponin IVb (**21**), clinoposaponin D (**24**), 11*α*,16*β*,23,28-tetrahydroxyolean-12-en-3*β*-yl-[*β*-D-glucopyranosyl-(1→2)]-[*β*-D-glucopyranosyl-(1→3)]-*β*-D-fucopyranoside (**25**), and buddlejasaponin IV (**48**)	(1) Platelet aggregation (%): 27.2 ±1.7 (**24**), 8.6 ± 1.2 (**21**), 5.0 ± 0.7 (**55**), 69.9 ± 1.8 (**48**), 2.0 ± 0.5 (**39**), 11.4 ± 1.2 (**25**), 74.1 ± 2.6 (**14**), 5.0 ± 0.7 (**13**), and 9.0 ± 1.1 (**45**) at 100 μM; ADP at 10 µM: 69.88±1.65 (positive control), polyphyllins II at 100 μM: 44.5 ± 5.5 (positive control). (2) EC_50_ of platelet aggregation activity: 53.4 μM (**48**), 12.2 μM (**14**), and other compounds (>100 μM). (3) Compounds **21** and **13** remarkably shortened TT by 20.6 and 25.1% at 200 μM, respectively. (4) Promoting effects on platelet aggregation and shortened TT.	[11]
	*C. chinense*	Ethanol extract	(1) Platelet adhesion rate 54.7 ± 10.7%. (2) Plasma calcium rehydration time 77.5 ± 9.6 s. (3) Hemostasis time of rats with femoral vein bleeding mode: 56.7 ± 5.8 s; hemostasis time in rabbit ear artery bleeding mode: 160.0 ± 17.3 s. (4) Improve platelet adhesion rate, shorten plasma recovery–calcium time.	[78]
	*C. chinense*	Total extract of *C. chinense* (TEC) obtained by water extraction and alcohol precipitation	Reduce metrorrhagia volume, alleviate pathological injury and increase MVD to promote recovery of the endometrium; TEC could also increase the levels of TXB2 and the expression of VEGF, TGF-b, and decrease the levels of IL-6, TNF-a and the expression of MMP-2/9.	[79]
	*C. chinense*	Total extract of *C. chinense* (TEC), total saponins of *C. chinense* (TSC), and total flavonoids of *C. chinense* (TFC).	TEC, TSC, and TFC all show therapeutic effects on AUB, particularly TEC. TSC exerts the effects by enhancing the coagulation function and promoting endometrial repair, and TFC by regulating estrogen levels and reducing inflammatory response.	[80]
	*C. chinense*	Total extract, buddlejasaponin IVb (**21**), hesperidin (**97**), naringenin (**93**), apigenin (**79**), and saikosaponin a (**46**) from *C. chinense*	(1) Hesperidin (**97**) and buddlejasaponin IVb (**21**) exerted a hemostatic effect. (2) Hesperidin (**97**), apigenin (**79**), naringenin (**93**), and saikosaponin a (**46**) promoted the proliferation of HEECs damaged by LPS, while buddlejasaponin IVb (**21**) did not significantly affect HEEC proliferation. (3) Significantly reduced the uterine bleeding volume, alleviated endometrial injury, increased plasma TXB2 level, and decreased plasma IL-6 and TNF-α levels.	[71]
Anti-cardiomyocyte damage and cardiovascular protection	*C. chinense*	Different polar fractions in total flavonoids	Inhibitory effect on the decrease in H9c2 cardiomyocyte viability.Increase SOD, GSH-Px, CAT activity, reduce LDH, MDA content.	[81]
	*C. chinense*	40% and 70% ethanol fractions of the total flavones	TFCC suppressed DOX-induced overexpression of p53 and phosphorylation of JNK, p38, and ERK. Studies with LY294002 (a PI3K/AKT inhibitor) demonstrated that the mechanism of TFCC-induced cardioprotection also involves activation of PI3K/AKT.	[82]
	*C. chinense*	Total flavonoids	(1) Prevented ISO-induced myocardial damage, including the decrease in serum cardiac enzymes and cardiomyocyte apoptotic index and improvement in the heart rate and vacuolation. TFCC also improved the free radical scavenging and antioxidant potential, thereby suggesting that one possible mechanism of TFCC-induced cardio protection is mediated by blocking oxidative stress. (2) TFCC pretreatment prevented apoptosis, increased the expression of HO-1, and enhanced the nuclear translocation of Nrf2. TFCC also activated phosphorylation of AKT, whereas the addition of LY294002, which is the pharmacologic inhibitor of PI3K, blocked the TFCC-induced Nrf2/HO-1 activation and cytoprotective effect.	[83]
	*C. chinense*	Total flavonoids	The expression levels of P21 and caspase-3 were reduced and the cell survival rate was increased, while the apoptosis rate, the MDA content, and the LDH activity were reduced. The CAT and SOD activities were increased. Inhibition of miR-702-5p inhibited hypoxia/reoxygenation-induced cardiomyocyte injury.	[84]
	*C. chinense*	Apigenin (**79**), luteolin (**80**), buddleoside (**81**), naringenin (**93**), eriodictyol (**94**), isosakuranetin (**95**), didymin (**96**), hesperidin (**97**), ethyl (2*R*)-3-(3,4-dihydroxyphenyl)-2-hydroxypropanoate (**110**), ethyl (2*E*)-3-(3,4-dihydroxyphenyl) prop-2-enoate (**112**), caffeic acid (**113**), ethyl (2*E*)-3-(2,3,4-trihydroxyphenyl) prop-2-enoate (**114**), ethyl rosmarinate (**116**), and clinopodic acid B (**117**)	Approximate EC_50_ of cell viability in high glucose-treated HUVECs: 11 μM (**80**), 8 μM (**93**), 19 μM (**94**), 3 μM (**110**), 36 μM (**113**), 4 μM (**116**), and 17 μM (**117**), 47 (Vit C, model). Other compounds (**79**, **81**, **95**, **96**, **97**, **110**, **112**, and **114**) showed weak protective effects (>60 μM).	[25]
	*C. chinense*	Clinopoditerpenes B (**26**) and C (**27**)	Cell viability: 73.7 ± 3.9% (**26**) at 12.5 μg mL^−1^, 64.6 ± 3.0% (H_2_O_2_-treated, model).	[14]
	*C. chinense*	Prunin (**98**)	Cell viability: exhibited viabilities of 84.25±7.36% (**98**) at 25.0 mg·mL^−1^, 62.12 ± 6.18% (model).	[26]
	*C. chinense*	Clinopodiside X (**3**); clinopodiside XI (**4**): clinoposaponin XIX (**42**)	Cell viability: 78.46 ± 1.47 (**3**), 80.77 ± 2.30 (**4**), 79.55 ± 1.85% (**42**), 64.19 ± 2.01% (model) at 50.0 μg·mL^−1^.	[8]
	*C. chinense*	Clinoposides G (**64**) and H (**65**)	(1) Cell viability of clinoposide G (**64**): 76.44 ± 2.75% (5 μg·mL^−1^), 81.25 ± 4.29% (10 μg·mL^−1^), and 87.66 ± 4.13% (20 μg·mL^−1^). (2) Cell viability of clinoposide H (**65**): 72.62 ± 3.51% (5 μg·mL^−1^), 77.89 ± 2.58% (10 μg·mL^−1^), and 85.62 ± 5.37 (20 μg·mL^−1^).	[21]
	*C. chinense*	Tournefolic acid B (TAB, **126**)	Tournefolic acid B (**126**) significantly improved the hemodynamic parameters (LVeDP, LVSP, +dP/dtmax, −dP/dtmin, and HR) of isolated rat hearts, and depressed the cardiomyocyte apoptosis. Furthermore, TAB inhibited the oxidative stress by adjusting the activities of antioxidant enzymes (SOD, CAT, and GSH-Px). The I/ R injury triggered endoplasmic reticulum (ER) stress by activating the ER proteins, such as Grp78, ATF6, PERK, and eIf2α, which are all refrained by TAB. TAB also enhanced the phosphorylation of PI3K and AKT, inhibited the expression of CHOP and Caspase-12, reduced the phosphorylation of JNK, and increased the Bcl-2/Bax ratio.	[27]
	*C. polycephalum*	Clinopodiside VI (**6**), saikosaponin c (**18**), arjunglucoside I (**74**)	Cell viability: 77.8 ± 2.6% (**6**), 80.9 ± 4.4% (**18**), 79.8 ± 2.7% (**74**), 63.3 ± 2.4% (model) at 100.0 μg·mL^−1^.	[9]
	*C. tomentosum*	Ethanolic extract	A significant proliferative effect of pAEC was observed at the highest dose. cTEE treatment was able to rescue LPS-induced injury. cTEE resulted in a significant increase in the migration and test tube formation capacity of pAEC. Quantitative PCR data showed a significant increase in FLK-1 mRNA expression.	[85]
	*C. vulgare*	Aqueous extract	Reduced the biomarkers of oxidative stress, including glutathione (GSH), malonedialdehyde (MDA), superoxide dismutase (SOD), glutathione peroxidase (GPx) and catalase (CAT); slightly decreased the systolic blood pressure by 20%.	[86]
Anti-inflammatory effect	*C. chinense*	Ethyl acetate fractions partitioned from 80% ethanol (CCE)	CCE suppresses PA-induced TLR4 expression in HUVECs, inhibiting downstream adaptor proteins (MyD88, TRIF, TRAF6) and blocking phosphorylation of IKKβ, NF-κB, JNK, ERK, and p38 MAPK, thereby reducing TNF-α, IL-1β, and IL-6 release. CCE also improves insulin signaling by reducing IRS-1 serine phosphorylation and enhancing tyrosine phosphorylation, restoring Akt/eNOS activation, and increasing NO production in PA-treated HUVECs. Additionally, CCE reverses impaired insulin-mediated vasodilation and eNOS function in rat aortas, suggesting CCE mitigates inflammation and insulin resistance by targeting TLR4-mediated NF-κB/MAPK pathways.	[87]
	*C. chinense*	Ethanol extract	(1) Inhibited inflammation by LPS-TLR4-NF-κB-iNOS/COX-2 signaling pathway in RAW264.7 cells. (2) Significantly alleviated pathological features with increased body weight and colonic length, decreased DAI and oxidative damage, and mediated inflammatory factors like NO, PGE2, IL-6, IL-10, and TNF-α.	[88]
	*C. gracile*	Aqueous extract and 95% ethanol extract	(1) Effectively reduce the twisting caused by acetic acid, raise the pain threshold of hot-plate mice, and inhibit the phase I and phase II reaction of mice treated by formalin. (2) Inhibit the significant degree of swelling of the ear caused by xylene in mice and inhibit the increase in the permeability of capillary wall caused by acetic acid. Significantly reduce the levels of NO, MDA, PGE2, IL-6, and TNF-α in the brain tissue and serum of mice.	[89]
	*C. polycephalum*	Imbricatusol I (**66**), saturol I (**67**), 3β-22, 25-dihydroxy-tirucalla-7,23-diene (**68**), maslinic acid (**69**), 2α, 3α-Dihydroxyolean-12-en-28-oic acid (**70**), hederagenin (**71**), 2α, 3α-dihydroxyursolic acid (**72**), alphitolic acid (**73**)	Inhibit the productions of NO in LPS-induced RAW 264.7 cells.	[22]
Antimicrobial and antibacterial activity	*C. bolivianum*	Ethanol extract	Decreased the uroplakin 1a expression and *E. coli* adhesion and invasion of uroepithelial cells while up-regulating caveolin-1.	[90]
	*Calamintha baborensis*	Hexanoic and chloroformic fractions from hydroalcoholic extract	The antibacterial activity of extracts showed good results with hexanoic and chloroformic fractions against *E. coli* (19 mm and 19.2 mm diameter of inhibition zone and MIC values about 43 and 43.4 μg·mL^−1^, respectively).	[46]
	*C. brevicalyx*	Essential oil	MIC: 125 μL·mL^−1^.	[44]
	*C. brownei*	Essential oil	Inhibitory concentrations ranging from 13.6 mg·mL^−1^ for *Staphylococcus epidermidis* ATCC 14990 to 3.1 mg·mL^−1^ for *Candida albicans* ATCC 10231.	[45]
	*C. menthifolium*	Essential oils from three Tunisian regions	Exhibited the highest fungitoxic properties toward *A. terreus* mold, *M. canis* dermatophyte, and *C. albicans* yeast (MIC values ranged from 40 to 400 μg mL^−1^).	[55]
	*C. macrostemum*	Essential oil	A remarkable antimicrobial activity on *Erwinia carotovora* (0.145), *Agrobacterium tumefaciens* (0.149), *Clavibacter michiganensis* (0.184), *Pseudomonas syringae* pv. phaseolitica (0.381), *Pseudomonas syringae* pv. Glycinea (0.437), *Escherichia coli* strain DH5a (0.515), *Fusarium oxysporum* (2.3), *Aspergillus niger* (2.9) and *Rhizopus stolonifer* (3.6).	[54]
	*C. nepeta*	Essential oil	The highest activity was found against *S. typhimurium* (1250 µg·mL^−1^). The essential oil is more effective against *B. cereus* (2500 µg·mL^−1^) and *S. sanguinis* (2500 µg·mL^−1^). The lowest activities were determined against *E. coli* (5000 µg·mL^−1^) and *P. aeruginosa* (10,000 µg·mL^−1^).	[62]
	*C. nepeta*	Essential oil	0.966 ± 0.057 µL·mL^−1^.	[91]
	*C. nepeta*	Ethyl acetate (AcOEt) extract, *n*-butanol (BuOH) extract, and dichloromethane (DCM) extract	*P. aeruginosa*: 100 14.89 ± 0.40 µg·mL^−1^ (DCM extract), 35.42 ± 1.00 µg·mL^−1^ (AcOEt extract), and 08.27 ± 3.11 µg·mL^−1^ (BuOH extract).	[92]
	*S. Calamintha Spp. Nepeta*	Essential oil	All tested molds were inhibited with 1/100 and 1/250 (*v*/*v*) concentrations after seven days of incubation. The minimum inhibitory and fungicidal concentrations of EO were in the orders of 0.666–2.666 μL·mL^−1^ and 2.666–5.333 μL·mL^−1^, respectively.	[61]
	*S. calamintha ssp. nepeta*	Essential oil	MIC ranged from 0.09 to 1.56 µL·mL^−1^.	[93]
	*C. sericeum*	Essential oil	Displays antibacterial activity against Gram-negative and Gram-positive bacterial strains (MIC 50–200 µg·mL^−1^) in a dose range close to standard antibiotics: 6–7 mm in Gram-positive bacterial strains, while Gram-negative bacteria have an inhibitory diameter of 6–8 mm.	[69]
	*S. calamintha* (L.) *Scheel.*	Essential oil	(1) MIC for bacteria was 0.007% (*v*/*v*) against *Enterococcus faecalis* and *Klebsiella pneumoniae*, whereas for fungi, it was 0.500% (*v*/*v*) against *Candida albicans*. (2) *Enterococcus faecalis* and *Listeria innocua* had the lowest minimum bactericidal concentration (MBC) at 0.125% (*v*/*v*), in contrast to the lowest fungicidal concentration (MFC) for Candida albicans at 0.500% (*v*/*v*).	[50]
Anti-cancer effect	*C. chinense*	Clinopoditerpene D (**28**), perovskiaditerpenoside B (**29**), 3*β*-hydroxy-12-*O*-*β*-D-glucopyranosyl-8,11,13-abietatrien-7-one (**30**), 12-*O*-*β*-D-glucopyranosyl-3,11,16-trihydroxyabieta-8,11,13-triene (**31**), cussoracoside A (**32**), clinoposaponin E (**17**), buddlejasaponin IVa (**55**), and clinopodiside G (**37**)	None of the compounds were cytotoxic (IC_50_ > 100 μM) against the A549 and HepG2 cancer cell lines.	[13]
	*C. chinense*	Clinopoursaponins A–D (**49**–**52**), clinopodisides VII–XII (**7**, **1**–**4**, **11**), saikosaponin g (**8**), 16*β*,23,28-trihydroxyoleana-9(11),12(13)-dien-3-yl-[*β*-D-glucopyranosyl-(1→2)]-[*β*-D-glucopyranosyl (1→3)]-*β*-D-fucopyranoside (**10**), 16*β*,21*β*,23,28-tetrahydroxyoleana-9(11),12(13)-diene-3-yl-[*β*-D-glucopyranosyl-(1→2)]-[*β*-D-glucopyranosyl-(1→3)]-*β*-D-fucopyranoside (**5**), 16*β*,23,28-trihydroxyoleana-9(11),12(13)-diene-3-yl-[*β*-D-glucopyranosyl-(1→4)-*β*-D-glucopyranosyl-(1→6)-*β*-D-glucopyranosyl-(1→3)]-[*β*-D-glucopyranosyl-(1→2)]-*β*-D-fucopyranoside (**12**), buddlejasaponin IV (**48**), clinoposaponin XVI (**40**), clinoposaponin XX (**41**), clinoposaponin XIX (**42**)	IC_50_ values (μM): 7.4 (**49**), 43.4 (**50**), 96.4 (**51**), 102.7 (**52**), 55.4 (**7**), 78.4 (compound **1**), 36.3 (**2**), 88.6 (**3**), 65.9 (**4**), 57.7 (**11**), >150 (**8**), 73.7 (**10**), 46.5 (**5**), 51.4 (**12**), 86.5 (**48**), 117.2 (**40**), 21.6 (**41**), 69.1 μM (**42**) and 7.6 μM (positive control, 10-hydroxycamptothecin) on 4T1 cells.	[8]
	*C. sericeum*	Essential oil	IC_50_ values: 213.40 ± 4.14 μM on T24, 202.50 ± 0.18 μM on DU-145, 197.80 ± 5.19 μM on MCF-7, and 195.90 ± 7.46 μM on HEK-293.	[69]
	*Calamintha incana*	Ethanolic extract	IC_50_ values: 260.20 ± 0.1 mg·mL^−1^ (Ethanolic extract), 0.23 ± 0.2 mg·mL^−1^ (Doxorubicin) on HepG2 cells.	[72]
	*C. umbrosum*	Petroleum ether, chloroform, and methanol extracts, buddlejasaponin IVa (**55**), and buddlejasaponin IV (**48**)	(1) IC_50_ values: >250 (petroleum ether extract), >167 (chloroform extract), and 239.5 μg·mL^−1^ (methanol extract) on the HN-5 cell line. (2) IC_50_ values: 472.7 (**55**), 19.1 μg·mL^−1^ (**48**) on HN-5 cells. (3) IC_50_ values: >500 (**55**), 18.6 μg·mL^−1^ (**48**) on HUVEC cells.	[94]
Antioxidant effect	*C. bolivianum*	Water, 65% ethanol, and 65% methanol	Antioxidant capacity: 725.9 ± 60.1 µmol TE/g (water extract), 908.2 ± 53.6 µmol TE/g (65% ethanol extract), 891.2 ± 41.5 µmol TE/g (65% methanol extract).	[95]
	*Calamintha baborensis*	EtOAc and *n*-BuOH extracts	(1) ABTS inhibition rate: 68.9% (EtOAc extract), 81.7% (*n*-BuOH extract). (2) DPPH: 27.6% (EtOAc extract), 80.99% (*n*-BuOH extract). (3) ORAC: 37.28% (EtOAc extract), 28.47 (*n*-BuOH extract). (4) FRAP: 21.73% (EtOAc extract), 19.52 μM·mL^−1^ (*n*-BuOH extract). (5) IC_50_ values: 23 ppm (EtOAc extract), 53.5 ppm (*n*-BuOH extract).	[46]
	*C. brownei*	Essential oil	IC_50_ (DPPH) 1.77 mg·mL^−1^, IC_50_ (ABTS) 0.06 mg·mL^−1^.	[45]
	*C. incana*	Essential oil	(1) ABTS: 129.58 ± 2.21 mg TEs/g oil. (2) CUPRAC: 51.14 ± 0.05 mg TEs/g oil. (3) FRAP: 53.63 ± 0.10 mg TEs/g oil.	[52]
	*Calamintha nepeta*	Essential oil	Interaction with the stable free radical of DPPH: 17.1% (20 min), 54.7% (60 min).	[96]
	*S. calamintha nepeta*	Essential oils extracted from wild *S. calamintha* (EOSS) and domesticated *S. calamintha* (EOSD)	(1) IC_50_ values of DPPH assay: 23.03 ± 4.30 (EOSS), 24.09 ± 4.38 µg/mL (EOSD). (2) EC_50_ values of FRAP assay: 55.38 ± 2.16 (EOSS), 60.72 ± 7.71 µg·mL^−1^ (EOSD).	[97]
	*C. nepeta*	AcOEt extract, *n*-butanol (BuOH) extract, and dichloromethane (DCM) extract	(1) IC_50_ value of DPPH: 8.12 ± 0.11 µg·mL^−1^ (BuOH extract). (2) IC_50_ value of ABTS^•+^ assay: 9.56 ± 1.12 µg·mL^−1^ (DCM extract). (3) IC_50_ value of GOR assay: 10.07 ± 0.40 µg·mL^−1^ (BuOH extract). (4) A_0.50_ of CUPRAC assay: 29.44 ± 0.65 µg·mL^−1^ (BuOH extract). (5) A_0.50_ of the phenanthroline assay: 9.85 *±* 0.07 µg·mL^−1^ (BuOH extract). (6) A_0.50_ of FRAP: 17.42 *±* 0.25 µg·mL^−1^ (BuOH extract).	[92]
	*C. serpyllifolium*	EtOH and dH_2_O extracts of stem and flower parts	(1) DPPH free radical scavenging activity: 92.14 ± 2.03% (EtOH extract of flower).ABTS cation scavenging assay: 89.2% (dH_2_O extract of flower). (2) FRAP value: 3.138 ± 0.08 (EtOH extract of flower). (3) CUPRAC value: 2.207 ± 0.92 (EtOH extract of stem), 2.061 ± 0.43 (dH_2_O extract of stem).	[98]
	*Calamintha incana*	Ethanolic extract	(1) IC_50_ values of DPPH: 35.9 ± 0.1 (ethanolic extract), 19.6 ± 0.2 µg·mL^−1^ (ascorbic acid, positive control). (2) IC_50_ of Reducing Power Assay: 90.3 ± 0.5 µg·mL^−1^ (ethanolic extract), 26.4 ± 0.2 µg·mL^−1^ (ascorbic acid, positive control).	[72]
	*C. sericeum*	Essential oil	(1) FRAP: 1.40 ± 0.05 mg TEAC/mL. (2) CUPRAC: 30.17 ± 1.60 mg TEAC/mL. (3) IC_50_ (ABTS): 106.06 ± 7.92. (4) IC_50_ (DPPH): 473.03 ± 14.11.	[69]
	*C. vulgare*	Acetone, methanol, and water extracts	(1) DPPH free radical scavenging activity: 81.72 mg TEs/g extract (water extract). (2) ABTS cation scavenging assay: 51.45 mg TEs/g extract (methanol extract). (3) CUPRAC assay: 44.32 (methanol extract). (4) FRAP assay: 87.25 (methanol extract).	[99]
	*C. vulgare*	CV3: fraction from *C. vulgare* extract by an RP 18 reversed-phase column	IC_50_ values of Fraction CV3: 0.02 mg·mL^−1^ (DPPH) and 0.0002 mg·mL^−1^ (ABTS), as well as the strongest ferric reducing potential (FRAP) of 0.89 mM TE/mg dw.	[100]
	*C. vulgare*	Aqueous and methanolic extract	(1) DPPH: 32.4 (aqueous extract), 25.7 μg·mL^−1^ (methanolic extract). (2) FRAP: 976.6 ± 17.1 μmol Fe^2+^/g of extract (aqueous extract), 2115.6 ± 99.4 μmol Fe^2+^/g of extract (methanolic extract).	[101]
Antihypertensive effect	*Calamintha vulgaris*	Crude extract and *n*-Hexane, chloroform, ethylacetate, and aqueous fractions	(1) Crude extract and fractions induced a fall in MAP in normotensive and high salt-induced hypertensive rats at different doses. The effect was more significant in the hypertensive rats (max. fall, 38.67 ± 2.17 vs 44.16 ± 4.67 mmHg). Among the fractions, chloroform was more effective (max. fall, 53.20 ± 1.23 mmHg) and aqueous the least (max. fall, 38.66 ± 1.12 mmHg); (2) The antihypertensive effect of *C. vulgaris* is the outcome of vasodilation, which is mediated through a combination of muscarinic receptor-linked NO, activation of TEA-sensitive K+ channels, prostacyclin and Ca^2+^ antagonism.	[102]
	*Calamintha officinalis*	Aqueous extract	(1) Reduced the systolic, diastolic, and mean arterial blood pressure in hypertensive rats. (2) Exerts a vasorelaxant ability through the sGC-cGMP induction pathway, vascular cyclooxygenase pathway, and the opening of K^+^ channels.	[103]
Enzyme inhibitory activity	*Calamintha incana*	Ethanolic extract	(1) α-Amylase Inhibition Assay: At the highest concentration investigated (100 μg·mL^−1^), the extract exhibited a noticeable effect on *α*-amylase by 88.5%. IC_50_ 33.5 ± 0.1 mg·mL^−1^ (acarbose, positive control), IC_50_ 46.3 ± 0.2 mg·mL^−1^ (ethanolic extract); (2) α-Glucosidase Inhibition Assay: The extract showed a noticeable effect on α-glucosidase activity at the highest concentration (100 μg·mL^−1^) of 70.5%. IC_50_ 37.1 ± 0.2 mg·mL^−1^ (epigallocatechin gallate, positive control), 56.8 ± 0.1 mg·mL^−1^ (ethanolic extract). (3) Pancreatic Lipase Inhibition Assay: The greatest levels of inhibition were 8.1% (ethanolic extract) and 92.2% (orlistat, positive control) at the same dose (100 mg·mL^−1^). (4) Dipeptidyl Peptidase-IV (DPP-IV) Inhibition Assay: The ethanolic extract of *C. incana* did not exert any considerable inhibition at any of the assessed concentrations compared to the positive control (sitagliptin), which displayed 95.2% inhibition of DPP-IV activity at the highest concentration (100 mg·mL^−1^).	[72]
	*C. chinense*	Apigenin (**79**), luteolin (**80**), buddleoside (**81**), naringenin (**93**), eriodictyol (**94**), isosakuranetin (**95**), didymin (**96**), hesperidin (**97**), ethyl (2*R*)-3-(3,4-dihydroxyphenyl)-2-hydroxypropanoate (**110**), ethyl (2*E*)-3-(3,4-dihydroxyphenyl) prop-2-enoate (**112**), caffeic acid (**113**), ethyl (2*E*)-3-(2,3,4-trihydroxyphenyl) prop-2-enoate (**114**), ethyl rosmarinate (**116**), and clinopodic acid B (**117**)	IC_50_ value of α-Glucosidase activity: 15.4 ± 2.3 μM (**79**), 2.0 ± 1.8 μM (**80**), 14.6 ± 2.2 μM (**81**), 57.1 ± 2.1 μM (**93**), 1.4 ± 3.4 μM (**94**), 31.2 ± 2.8 μM (**95**), > 60 μM (**96**, **97**, and **113**), 18.5 ± 2.9 μM (**110**), 17.8 ± 1.8 μM (**112**), 12.0 ± 2.3 μM (**114**), 1.2 ± 4.8 μM (**116**), and 0.6 ± 2.8 μM (**117**).	[25]
	*C. nepeta*	AcOEt extract, *n*-butanol (BuOH) extract, and dichloromethane (DCM) extract	(1) IC_50_ value of AChE assay: 170.1 ± 1.58 µg·mL^−1^ (DCM extract). (2) IC_50_ values of BChE assay: 73.06 ± 0.83 µg·mL^−1^ (DCM extract), 187.8 ± 1.57 µg·mL^−1^ (AcOEt extract).	[92]
	*C. nepeta*	EO and aqueous extracts	(1) AChE IC_50_ (μg·mL^−1^): 205.6 ± 10.3 (EO); 983.9 ± 49.2 (aqueous extracts). (2) BChE IC_50_ (μg·mL^−1^): 88.3 ± 4.4 (EO); 1669.9 ± 83.5 (aqueous extracts).	[104]
	*C. serpyllifolium*	EtOH extract of flower	AChE inhibition percentage: 60.18 ± 1.37%, BChE: 72.15 ± 0.98%, and TYR 55.04 ± 2.04% at 1000 μg·mL^−1^.	[98]
	*C. vulgare*	Acetone extract	(1) Acetylcholinesterase: 1.34 ± 0.01 mg GALAEs/g extract. (2) Butyrylcholinesterase: 0.93 ± 0.23 mg KAEs/g extract. (3) Tyrosinase: 1.85 ± 0.40 mg KAEs/g extract.	[99]
	*C. vulgare*	Methanol and water extracts	(1) α-Amylase: 0.70 ± 0.03 (methanol extract), 0.10 ± 0.01 (water extract); (2) α-Glucosidase: 1.82 ± 0.27 (methanol extract), 3.54 ± 0.01 (water extract).	[99]
	*C. vulgare*	CV3: fractions from *C. vulgare* extract by an RP 18 reversed-phase column	CV3 showed moderate *α*-glucosidase and *α*-amylase inhibitory potential.	[100]
	*C. vulgare*	Aqueous extract and methanolic extract	AChE enzyme: 14.6 ± 2.1% (aqueous extract), 6.3 ± 1.1% (methanolic extract).	[101]

## Data Availability

The data presented in this study are available on request from the authors.
